# Comparative Analysis of *WRKY* Genes Potentially Involved in Salt Stress Responses in *Triticum turgidum L*. ssp. *durum*

**DOI:** 10.3389/fpls.2016.02034

**Published:** 2017-01-31

**Authors:** Fatma-Ezzahra Yousfi, Emna Makhloufi, William Marande, Abdel W. Ghorbel, Mondher Bouzayen, Hélène Bergès

**Affiliations:** ^1^Laboratory of Plant Molecular Physiology, Center of Biotechnology of Borj Cedria, Borj Cedria Science and Technology ParkHammam-lif, Tunisia; ^2^Centre National de Ressources Genomiques Vegetales, French Plant Genomic Center, INRA–CNRGVCastanet-Tolosan, France; ^3^INRA, UMR990 Genomique et Biotechnologie des FruitsCastanet-Tolosan, France; ^4^INPT, Laboratoire de Genomique et Biotechnologie des Fruits, University of ToulouseCastanet-Tolosan, France

**Keywords:** BAC sequencing, *cis*-regulatory elements, WRKY, transposable elements, salt stress, *Triticum durum*, wheat

## Abstract

WRKY transcription factors are involved in multiple aspects of plant growth, development and responses to biotic stresses. Although they have been found to play roles in regulating plant responses to environmental stresses, these roles still need to be explored, especially those pertaining to crops. Durum wheat is the second most widely produced cereal in the world. Complex, large and unsequenced genomes, in addition to a lack of genomic resources, hinder the molecular characterization of tolerance mechanisms. This paper describes the isolation and characterization of five *TdWRKY* genes from durum wheat (*Triticum turgidum L*. ssp. *durum*). A PCR-based screening of a *T. turgidum* BAC genomic library using primers within the conserved region of *WRKY* genes resulted in the isolation of five BAC clones. Following sequencing fully the five BACs, fine annotation through Triannot pipeline revealed 74.6% of the entire sequences as transposable elements and a 3.2% gene content with genes organized as islands within oceans of TEs. Each BAC clone harbored a *TdWRKY* gene. The study showed a very extensive conservation of genomic structure between TdWRKYs and their orthologs from Brachypodium, barley, and *T. aestivum*. The structural features of TdWRKY proteins suggested that they are novel members of the WRKY family in durum wheat. TdWRKY1/2/4, TdWRKY3, and TdWRKY5 belong to the group Ia, IIa, and IIc, respectively. Enrichment of *cis*-regulatory elements related to stress responses in the promoters of some *TdWRKY* genes indicated their potential roles in mediating plant responses to a wide variety of environmental stresses. *TdWRKY* genes displayed different expression patterns in response to salt stress that distinguishes two durum wheat genotypes with contrasting salt stress tolerance phenotypes. *TdWRKY* genes tended to react earlier with a down-regulation in sensitive genotype leaves and with an up-regulation in tolerant genotype leaves. The *TdWRKY* transcripts levels in roots increased in tolerant genotype compared to sensitive genotype. The present results indicate that these genes might play some functional role in the salt tolerance in durum wheat.

## Introduction

Durum wheat (*Triticum turgidum* ssp. *durum*) is a monocot of the *Poaceae* family, of the *Triticeae* tribe and the *Triticum* genus. Tetraploid *Triticum durum* (2*n* = 4× = 28, AABB), originated when two diploid wild grasses were crossed. The A genome originated from *T. urartu* (Dvorak, 1988). The origin of the B genome is still under discussion, but so far, *Ae. Speltoides* has been put forward as the closest to the donor of this genome (Fernandez-Calvin and Orellana, 1994). Durum wheat has one of the largest and most complex genomes; its size is estimated at 13,000 Mb.

The unavailability of *T. durum* genome sequences complicates and hinders the identification of the genetic factors and mechanisms behind responses to abiotic stresses such as drought and salinity. These are just some of the major constraints affecting cereal crops. In terms of commercial production and human food, this species is the second largest of its kind after bread wheat (*Triticum aestivum* L.). Durum wheat represents 8% of total wheat production, but 80% grow under Mediterranean climates (Monneveux et al., 2000). To date, durum wheat is the only tetraploid wheat species of commercial importance that is widely cultivated in these regions, where drought, heat and salinity limit yield considerably. Durum wheat is also more salt sensitive than bread wheat (Gorham et al., 1990) and saline soil has a negative effect on production (Maas and Grieve, 1990). Consequently, special efforts must be made to increase its tolerance.

Plants adapt to adverse environmental conditions through the induction of stress-responsive and stress-tolerant genes, a process that occurs mainly through transcription factors (TFs). TFs are known to mediate stress signal transduction pathways regulating downstream target gene expression and lead to stress tolerance (Shinozaki and Dennis, 2003; Chen and Zhu, 2004; Yamaguchi-Shinozaki and Shinozaki, 2005; Budak et al., 2013).

The WRKY transcription factor belongs to a very large family of transcription factors potentially involved in drought/salt stress response (Budak et al., 2013). This family originated in early eukaryotes and greatly expanded in plants (Zhang and Wang, 2005). It counts over 70 members in the Arabidopsis genus (*Arabidopsis thaliana*; Eulgem et al., 2000; Dong et al., 2003), 55 members among cucumber plants (*Cucumis sativus*; Wei et al., 2012), 119 members among maize plants (*Zea Mays*; Ling et al., 2011), 94 members among barley plants (*Hordeum vulgare*; Liu et al., 2014), 182 encoding genes in soybean (Glycine max; Bencke-Malato et al., 2014), 62 members among diploid woodland strawberry plants (*Fragaria vesca*; Wei et al., 2016) and 100 members among rice grasses (*Oryza sativa*; Xie et al., 2005).

This transcription factors family is characterized by a 60 amino acids domain containing the WRKY amino acid sequence at its amino-terminal end and a putative zinc finger motif at its carboxy-terminal end. It binds specifically to the (T)(T)TGAC(C/T) sequence motif, known as the W box, which requires both the invariable WRKY amino-acid signature and the cysteine and histidine residues of the WRKY domain, which tetrahedrally coordinate a zinc atom (Rushton et al., 2010).

The existence of either one or two highly conserved WRKY domains is the most vital structural characteristic of the *TdWRKY* gene (Bi et al., 2016). Furthermore, the global structures of WRKY proteins are highly divergent and can be classified into different groups, which might reflect their distinct roles. WRKY proteins are classified into 3 main groups (I, II, III) based on the number of WRKY domains and the structure of the zinc finger-like-motif.

Group I proteins contain two WRKY domains followed by a C2H2 zinc finger motif. The other WRKY proteins from group II and III contain one WRKY domain followed by a C2H2 or C2HC accordingly. Group II can be divided into five subgroups (IIa, IIb, IIc, IId, and IIe) based on additional amino acid motifs (Yamasaki et al., 2005).

*WRKY* genes are known to participate in various developmental and physiological metabolisms, including disease resistance (Bhattarai et al., 2010), senescence (Besseau et al., 2012), growth and developmental processes (Guillaumie et al., 2010), as well as biotic and abiotic stress responses (Mingyu et al., 2012). Recently, transgenic Arabidopsis plants overexpressing *TaWRKY2* (*EU665425*) or *TaWRKY19* (*EU665430*) have shown improved tolerance to salt, drought and/or freezing stresses when compared with the wild-type plants (Niu et al., 2012). Marè et al. (2004) described *HvWRKY38* (*AY541586*), a barley gene coding for a WRKY protein, whose expression is involved in cold and drought stress response.

In durum wheat, very few *WRKY* EST sequences have been identified. Only partial *cDNA*s were found (Budak et al., 2013; Cifarelli et al., 2013). Literature does not include any report describing the characterization of a *TdWRKY* gene from tetraploid wheat species.

Available plant genome sequences from monocot plants are key resources that enable a better understanding of their gene content, structure and function. They are also indispensable for understanding transposable elements, intergenic space organization and composition. Genomic comparisons between the genome A diploid wheat donor (*Triticum urartu*), hexaploid wheat (*T. aestivum*, The International Wheat Genome Sequencing Consortium (IWGSC), 2014), barley (*H. vulgare*, The International Barley Sequencing Consortium, 2014) and Brachypodium (*Brachypodium distachyon*, The International Brachypodium Initiative, 2010), have not only confirmed the broad synteny between *Poaceae* gene content but have also helped to deduce their function within a phylogenetic context (Dubcovsky et al., 2001).

In this paper, we obtained genomic sequences of the region harboring *WRKY* genes from *T. turgidum* ssp. *durum* from Langdom#65 BAC clone library screening. First, we presented the *WRKY* gene characterization, including phylogenetic analysis and orthologous gene comparison. Then, we analyzed the genomic environment of *WRKY* genes, including intergenic space composition, and *cis*-acting elements that helped to associate a putative function with *TdWRKY*s. We finally performed *TdWRKYs* differential gene expression. *TdWRKY1, 3, 4*, and *5* were induced by high-salt treatment in two durum wheat varieties, Grecale (GR) and Om Rabiaa (OR), shown to be salt-tolerant and -sensitive, respectively, suggesting that *TdWRKYs* may be involved in salt-stress responses. These data provide new leads toward improving durum wheat tolerance to abiotic stresses.

## Materials and methods

### Plant material

Three genotypes of tetraploid *T. turgidum L*. ssp. *durum* (2*n* = 4× = 28) were used in this study; Langdon LDN#65 for PCR bacterial artificial chromosome (BAC) screening, and OR and GR for functional analyses. The latter two were a local Tunisian variety and an Italian variety introduced in Tunisia, respectively.

### BAC library screening

The BAC library constructed from the tetraploid *T. turgidum L*. ssp. *durum* (2*n* = 4× = 28) Langdon LDN#65 genotype was used in this study for PCR Bacterial Artificial Chromosome (BAC) screenings. The BAC library is represented by a total of 516,096 clones organized in 1,344,384-well plates. The average insert size of the BAC clones was estimated at around 131 kb resulting in 5.1 genome coverage (Cenci et al., 2003). The LDN#65 BAC library and related tools are available at the French Plant Genomic Resource Center upon request (http://cnrgv.toulouse.inra.fr/en/library/genomic_resource/Ttu-B-LDN65). The library was organized into a two-dimensional pool and BAC library screening was performed as described by Cenci et al. (2003, 2004). The pooling strategy used required 56 PCRs for the superpools (9216 BAC clones each) and 50 additional PCRs for each positive superpool. The screenings have been done as described in Makhloufi et al. (2014).

#### PCR primer design and PCR amplification for BAC screening

Two primer design strategies have been used. *O. sativa, T. aestivum* and *H. vulgare* sequences harboring conserved WRKY domains were used as queries (NCBI: http://www.ncbi.nlm.nih.gov/; GrainGenes: http://wheat.pw.usda.gov/GG2/index.shtml; Gramene: http://www.gramene.org/; TIGR: http://www.jcvi.org/; HarvEST: http://harvest.ucr.edu/; Phytozome:http://www.phytozome.net/; PTDB: http://plntfdb.bio.uni-potsdam.de/v3.0; and plant GDB: http://www.plantgdb.org/), in a BLAST search to find homologous *T. turgidum* ESTs (Budak et al., 2013; Cifarelli et al., 2013). Multiple alignments of the DNA sequences were performed by ClustalW software (Larkin et al., 2007). In order to avoid an amplification of the exon–intron junction, prediction of the exon boundaries within *Triticeae* expressed sequence tags (ESTs) were performed based on rice and Arabidopsis genomic sequences.

The second has been deduced due to the very strong homology between different species of *Triticeae*. We tried to identify non-specific *T. turgidum* primers from genes whose function was studied. For this, some *WRKY* genes that had been specifically studied in the context of abiotic stress, were chosen for primer design: *HvWRKY38* (*AY541586*) (group IIa) (Marè et al., 2004) and *TaWRKY2 (EU665425)* (group I); *TaWRKY19 (EU665430)* (group I) (Niu et al., 2012).

PCR primers were then designed to cover exons of the entire selected sequence using the Perl primer tool v.1.1.2.1 (Marshall, 2004) (Table S1). Primers were tested on genomic DNA of LDN#65 before BAC library screening. Total DNA was extracted from wheat Langdon 65 variety using the Plant DNAzol® reagent. PCR conditions used were as follows: initial denaturation at 95°C for 5 min, followed by 45 cycles of 20 s at 95°C, 16 s at 60°C, and 20 s at 72°C, performing a melting curve with an increment of 0.5°C per cycle. PCR products for the selected BACs were separated by electrophoresis (2% Agarose).

### BAC sequencing, assembly, and annotation

BAC DNA was extracted using a NucleoSpin® 96 Flash kit (Macherey-Nagel) and the insert size was estimated with NotI digestion (Fast Digest NotI; fermentas). Positive BAC clones were outsourced for 454 Life Sciences pyrosequencing technology using the GS Junior Roche system (Kit 454 Titanium; Roche). Sequence data assembly was performed with Newbler 2.8 software sold by 454 Life Sciences/Roche for 454 data (Veras et al., 2013). The assembly was performed on data previously processed by the software Pyrocleaner after clearing reads from contamination by the host *E. coli*. Sequenced BAC DNA was analyzed using TriAnnot Pipeline v.3.8 improved for wheat species (http://wheat-urgi.versailles.inra.fr/Tools/TriAnnot-Pipeline) enabling annotation, masking of transposable elements, and gene structure organization (Text S1) (Leroy et al., 2012).

### Alignment, phylogenetic tree, and sequence analysis of *TdWRKY* genes

The TdWRKY protein sequences were submitted to the CDD (Conserved Domains Database) from NCBI, and to Motif Scan detection in MyHits (http://myhits.isb-sib.ch/) with Prosite databases selected. Homologous proteins from whole genome sequenced monocot plants (Wheat, Barley, Brachypodium, Maize, Sorghum and Rice) and Arabidopsis were selected for sequence alignments using DNAMAN software (http://www.lynnon.com/).

A neighbor joining phylogenetic tree was derived from a MUSCLE alignment 3.8 (Edgar, 2004) of TdWRKY proteins, their homologous WRKY proteins from cereals and their closest members from *A. thaliana*. The tree was then produced by MEGA 6 software (Tamura et al., 2013) using the Neighbor-Joining method with 1000 bootstrap replicates.

A 1.5 kb DNA fragment on the 5′-regulatory region upstream of the transcription start of the *TdWRKY* gene was subjected to *in silico* analysis using the PLACE signal scan and NSITE-PL (Recognition of PLANT Regulatory motifs with statistics) from the Softberry tool (http://www.softberry.com/) to search for putative *cis-*regulatory elements in the promoter region potentially involved in controlling *TdWRKY* gene expression and also in the promoter region of their orthologs in *T. aestivum* and *H. vulgare* to see the average frequencies of *cis*-acting elements related to abiotic stress in these promoters.

### Salt stress

Sterile seeds of two independent genotypes from *T. turgidum* subsp. durum, OR and GR, were first stored for 48 h at 4°C for initialization of germination. Seedlings were sown in recipient Magenta™ vessels containing 50 ml of 50% MS-based medium (Murashige and Skoog, 1962) and were left for 10 d in an *in vitro* growth chamber maintained at a controlled photoperiod of 14 h during the day at 25°C with 80% humidity and an intense luminosity of 250 μmol m^−2^ s^−1^, and for 10 h during the night at 20°C. They were then subjected to abiotic and hormonal stress treatments. For salinity treatment, seedlings were transferred into 50% MS medium containing 200 mM NaCl for 6 or 24 h. Leaves and roots were then harvested separately, dropped immediately into liquid nitrogen, and stored at −80°C for RNA extraction.

### Gene expression analysis

Total RNA from at least 30 salt-treated and untreated leaves and roots from OR and GR genotypes were extracted using a Pure Link Plant RNA Reagent kit (Invitrogen). Total RNA was DNase treated (Promega), and first-strand cDNA was reverse transcribed from 2 μg of total RNA using an M-MLV Reverse Transcriptase kit (Promega) according to the manufacturer's instructions. First-strand *cDNA* generated from total *RNA* including salt-treated and untreated samples from either the OR or GR genotype was subjected to real time quantitative expression analysis. The latter was performed in a fluometric thermal cycler (DNA Engine Opticon 2; MJ Research, Walthan, MA, USA) using SYBR Green fluorescent dye following the manufacturer's instructions. Results were shown using SDS2.2 software on an Applied Biosystem 7900 HT Fast Real-Time PCR System. Comparisons of repeated samples were assessed using CT values among the three replications. Linear data were normalized to the mean CT of 26S rRNA as an internal reference gene and the relative expression ratio was calculated using the formula 2^−ΔΔCT^. Log2-transformed signal ratio was carried out. The gene specific primers used for PCR are listed in Table S2.

## Results

### Identification, sequencing and general features of the sequence composition of *T. durum* BAC clones

516,000 clones from the Durum wheat bacterial artificial chromosome (BAC) library, were screened for individual clones harboring *WRKY* genes. Six *T. durum* BAC clones were selected and fully sequenced by 454 technologies.

BAC clone annotation revealed 12 non-TE genic features that were classified into two categories: 9 protein coding genes, one hypothetical and 2 gene fragments (Table S4). The gene assignments were all supported by at least one full-length cDNA, an EST and/or homolog in another monocot plant such as *T. aestivum, O. sativa, H. vulgare, Z. mays*, and *B. distachyon*. One gene per insert was predicted for TD14H23 (GenBank accession no. KY091673), TD473J01(GenBank accession no. KY091677), TD493B21 (GenBank accession no. KY091678) and TD315C07 (GenBank accession no. KY091676). The remaining two BAC clones TD16L16 (GenBank accession no. KY091674) and TD789O23 (GenBank accession no. KY091679), contain four genes (Table 1). Two genes (of known or unknown function), three genes with putative function, four genes with domain-containing proteins, one hypothetical gene and one truncated (fragmented gene), were identified (Table 1, Table S4).

**Table 1:** **TdWRKY gene models and comparaisons with homologous sequences in grass species using Blastp and Blastn**.

		**BLASTP**					**BLASTn**				
**BAC clone ID**	***T.turgidum*** **predicted gene**	**Protein Acc.No**		**Identity (%)**	**Score**	**Expect**	**cDNA Acc.No**		**Identity (%)**	**Score**	***E*-value**
TD14H23	Known_function, WRKY DNA binding domain 3534bp 1407bp 469aa CAT01	*T. aestivum*	ACD80362.1	98	953	0.0	*T. aestivum*	EU665430.1	99	2521	0.0
		*T. urartu*	EMS57536.1	94	811	0.0	–	–	–	–	–
		*H. vulgare*	BAJ96672.1	78	755	0.0	*H. vulgare*	AK365469.1	93	1625	0.0
		*B. distachyon*	XP_003562765.1	70	635	0.0	*B. distachyon*	XM_003562717.3	86	1137	0.0
		*A. thaliana*	NP_178433.1	45	228	1e-67	–	–	–	–	–
TD16L16	Putative_function, Putative WRKY transcription WRKY 3052bp 1716bp 572aa CAT04	*T. urartu*	EMS51071.1	93	1124	0.0	*B. distachyon*	XM_010236958.1	86	522	5e-69
		*A. taushii*	EMT16491.1	88	1010	0.0	–	–	–	–	–
		*B. distachyon*	XP_010235260.1	53	536	2e-180	–	–	–	–	–
		*A. thaliana*	NP_200438.1	30	199	9e-55	–	–	–	–	–
TD493B21	Putative function, WRKY DNA binding domain 1227bp 882bp 294aa CAT01	*T. aestivum*	ABN43185.1	99	605	0.0	*T. aestivum*	EF368364.1	99	1591	0.0
		*T. aestivum*	AGF90798.1	98	602	0.0	*S. italica*	XM_004962359.3	79	503	3e-138
		*A. tauschii*	EMT33135.1	87	508	2e-176	*T. aestivum*	EF368357.1	92	1151	0.0
		*A. thaliana*	AAM34736.1	54	278	5e-89	*T. aestivum*	EU665443.1	91	1138	0.0
TD473J01	Putative_function, WRKY transcription factor 4295bp 1053bp 351aa CAT04	*T. aestivum*	AFW98256.1	100	724	0.0	*T. aestivum*	JX679079.1	99	1940	0.0
		*H. vulgare*	CAD60651.1	91	626	0.0	*H. vulgare*	AK360269.1	92	1487	0.0
		*S. purpurea*	AJF34891.1	81	554	0.0	*H. vulgare*	AY541586.1	94	1367	0.0
		*B. distachyon*	XP_003570741.1	70	437	3e-149	*B. distachyon*	XM_003570693.3	80	734	0.0
		*A. thaliana*	NP_178199.1	36	198	9e-60	–	–	–	–	–
TD315C07	Domain_containing_protein, WRKY DNA binding domain 2157bp 1107bp 369aa CAT02	*T. aestivum*	CDM83596.1	100	751	0.0	*H. vulgare*	AK358052.1	92	1507	0.0
		*H. vulgare*	BAJ98040.1	89	511	1e-177	*T. aestivum*	HG670306.1	99	1011	0.0
		*B. distachyon*	XP_003569516.1	70	356	1e-116	*B. distachyon*	XM_003569468.3	82	861	0.0
		*A. thaliana*	NP_174279.1	46	173	3e-50					
TD789O23	Gene fragment, WRKY DNA binding domain 683bp 402bp 134aa CAT04	*T. aestivum*	CDM83596.1	98	285	3e-85	*T. aestivum*	HG670306.1	93	990	0.0
		*H. vulgare*	BAJ98040.1	91	177	4e-51	*H. vulgare*	AK358052.1	92	785	9e-162
		*B. distachyon*	XP_003569516.1	74	100	4e-22	*B. distachyon*	XM_003569468.3	78	246	4e-61
	Gene fragment, WRKY DNA binding domain 1567bp 861bp 287aa CAT03	*T. aestivum*	CDM83596.1	98	375	1e-125	*H. vulgare*	AK358052.1	93	1016	0.0
		*H. vulgare*	BAJ98040.1	91	352	8e-117	*T. aestivum*	HG670306.1	94	1689	0.0
		*B. distachyon*	XP_003569516.1	74	273	2e-85	*B. distachyon*	XM_003569468.3	84	584	9e-163
		*A. thaliana*	NP_193551.1	75	174	5e-51	–	–	–	–	–

*The genes models were defined on the table using the genomic sequence length (bp), the CDS length (bp), the protein length (aa) and the categories (CAT) as a quality index for the annotator (CAT01, CAT02, and CAT03 correspond to biological evidence found on Triticum and Aegilops Full-length cDNAs, Poaceae Full-length cDNAs and CDS from O. sativa (IRGSP) and B. distachyon genomes annotation, respectively, the genes models predicted by EuGene belong to CAT04*.

An average GC content of 47.5% was found for all BACs. In contrast with the constant GC content, gene and TE composition was highly variable between the different BAC sequences. The proportion of TEs ranged from 64 to 91%, while gene content ranged from 1 to 4 genes per BAC. The coding fraction of the 960,769 kb total samples represents 3.2% of the sequences, while the TE content is 74.6%, distributed as follows: 63.6% for class I, 7.9% for class II and 3.1% for unclassified TEs (Table S3). While class I retrotransposons constitute the highest TE proportion of the 6 sequenced regions, BAC clone TD473J01 shows the highest proportion of *CACTA* class II (17.0%). Class I TE DNA sequences were distributed as follows: 27.2% *Gypsy*- (150 TEs) and 15.9% *Copia*- (71 TEs) like “long terminal repeats (LTR)”-retrotransposons. New class I transposable elements were identified for the first time in this study. They account for 17.9% of length and were identified as *de novo* LTR-retrotransposons (Table S5). Class II TEs (DNA transposons) represent 7.9% of the cumulative sequence length. The *CACTA* TEs represent the majority (32.4%) of class II DNA sequences. Novel class II TE families were identified, sharing weak homologies with known *CACTA, Mutator* and *Mariner*. The 3.1% novel unclassified elements, share a stretch of weak homology with other Triticeae unclassified transposable elements (Table S3, Text S2).

### *TdWRKY* and monocot gene structure

A comparison of durum wheat, *T. aestivum, H. vulgare*, and *Brachypodium distachyon WRKY* gene sequences helped to establish their structure.

**Figure 1:**
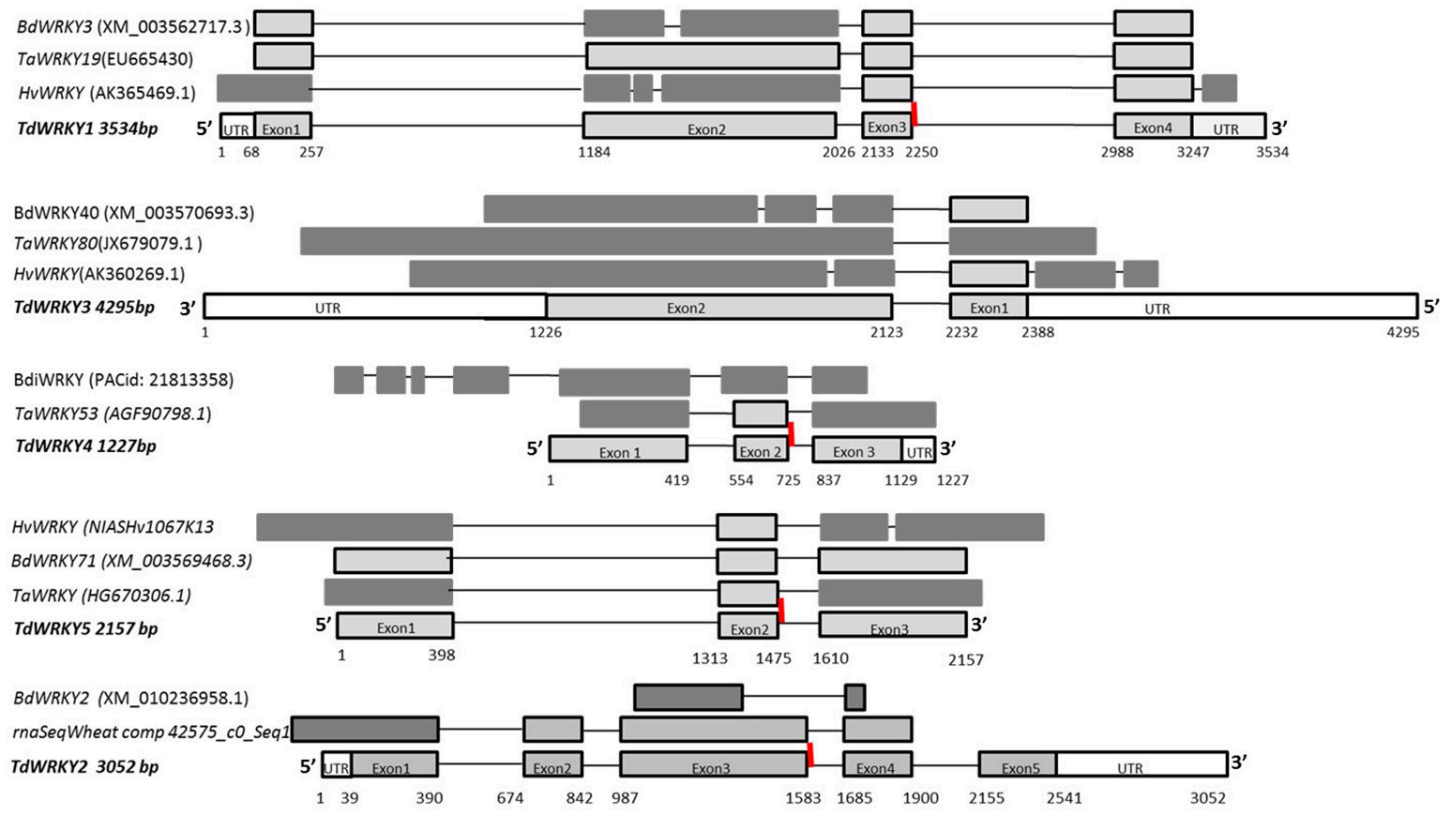
**Isolation of 5 *TdWRKY*s by screening of a durum wheat BAC library and Comparative Structure of *TdWRKY*s and orthologous from *B.distachyon*, *T. aestivum* and *H. vulagre***. Genomic structure of the durum wheat TdWRKYs gene; white portion are UTR regions, dark gray portions are orthologous exons from different sizes, and light gray boxes are exons from same size between orthologous. The line represents the intron. The red motives are special WRKY family conserved intron. *TdWRKYs* untranslated regions, introns and exons boundaries are highlighted on the bottom of *TdWRKYs*.

Gene length varies from 1.2 to 4.2 Kb. It is conserved amongst the orthologous monocots (*TdWRKY1* and *TdWRKY5* length was highly conserved among orthologous members). The number of introns varies from 2 to 4. The intron size is also variable; it ranges from 102 to 925 bp (Figure 1, Table 1). All exon-intron junction sites obey the GT/AG rule as identified in other eukaryotic genes. To date, the relative organization of the exons and introns is the same for the other *WRKY* genes characterized in cereal, i.e., the number of exons and introns remains the same and individual introns occur at relatively the same sites for barley, Brachypodium and wheat genes.

The size of coding sequences varies from 882 to 1716 bp (Figure 1). The size of exonic regions between the orthologous genes was similar although the overall region structure was slightly different between *TdWRKY*s and their orthologs (exon 2 from *TdWRKY1* and *TdWRKY2* were almost the same size, but with 1 to 3 more intron phases in corresponding exons. The same was observed with exon 3 from *TdWRKY5*). Small stretches of exonic region sequences or more exons (*BdiWRKY* has 4 more exons, in the 5′ moiety, than *TdWRKY4*) do not contradict the general pattern of overall high *WRKY* gene conservation between the *TdWRKY*s and their homologous genes (Text S3).

Comparison of the *TdWRKY*s cDNA with sequences of other species showed an identity ranging from 99% for *T. aestivum;* 86% for Brachypodium and 92% for barley (Table 1, Text S3).

Interestingly, we also identified a cluster of non syntenic collinear genes, probably originating from genomic rearrangements of gene blocks such as *gene 1* and *gene 4* from TD789O23 (the two gene fragments from Table 1). They were annotated as being similar to the WRKY domain protein. *Genes 1* and *4* (order on the BAC clone), share 93 and 94% of similarity, respectively, with *TdWRKY5*. An alignment of *TdWRKY5* from TD315C07 and the two fragments (*gene 1* and *gene 4*) from TD789O23, revealed that *gene 4* might be a truncated second allelic form of *TdWRKY5* (Figure 1). In fact, the *gene 4* genomic sequence is 989 bp shorter (from position 1 to 990, Figure S2) than *TdWRKY5*. It results in the formation of a new coding protein. The predicted protein from *gene 4* belongs to group II as well as *TdWRKY5*. The WRKY domain on exon2, the PR intron special feature, and the zinc finger-like motif on exon3, are perfectly conserved. The alignment of *TdWRKY5, gene 4* and *gene 1*, showed that *gene 1* and *gene 4* happened to be fragments of *TdWRKY5* (Figure S2). This disruption was the result of a deletion and an insertion. These are two of the most important genomic rearrangement events. They play significant roles in genome evolution. This reshuffling created two new, non-syntenic genes with orthologous species (Table 1). This might, or might not, be a loss-of-function mutation of the second *TdWRKY5* allelic form. Unfortunately, we were only able to find approximately 450 bp from the 898 bp of newly inserted fragment (*gene1*) since it is located at the extreme 5′ start of TD789O23 DNA insert. There are many ways in which exon shuffling may occur. Shuffling involves transposable elements such as *LINE-1* retroelements and *Helitron* transposons, as well as *CACTA* elements and *LTR* retroelements, a crossover during sexual recombination or alternative splicing. These hypothesized mechanisms should be thoroughly explored.

### TdWRKYs classification and phylogeny

**Figure 2:**
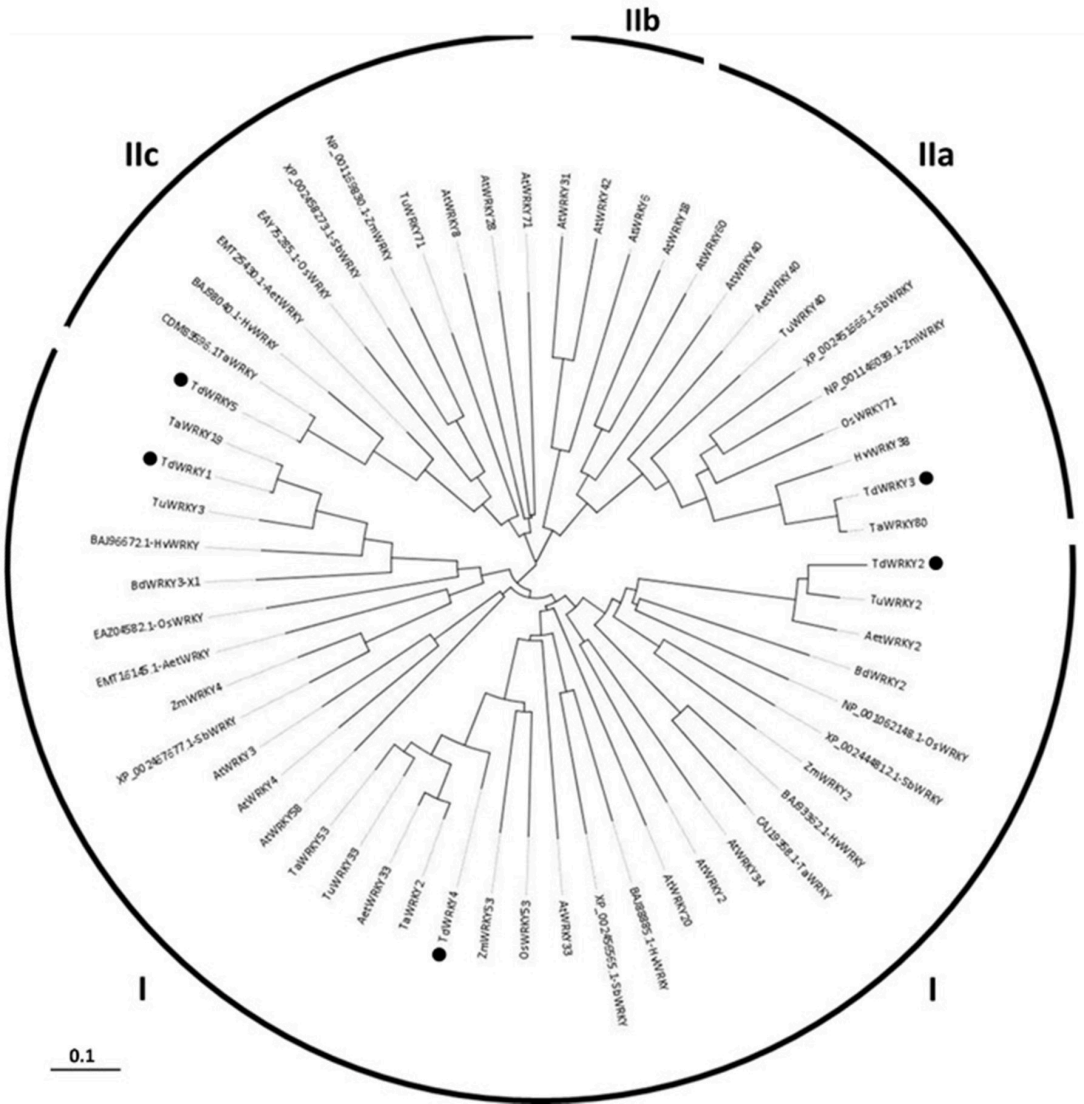
**Phylogenetic analysis of the TdWRKYs proteins with orthologous members from cereals (*T. aestivum*, *T. urartu*, *Ae. taushii*, *H. vulgare*, *Z. mays*, *S. bicolor*, *and O. sativa*) and *A. thaliana***. The Arabidopsis WRKY sequences were downloaded from GenBank (we used gene names for the alignment). TdWRKYs are spotted with black motives. Alignment of the 59 amino acids sequences was performed by MUSCLE 3.8 (http://www.ebi.ac.uk/Tools/msa/muscle/). The evolutionary history was inferred using the Neighbor-Joining method. The bootstrap consensus tree inferred from 1000 replicates is taken to represent the evolutionary history of the taxa analyzed. The evolutionary distances were computed using p-distance method and are in the units of the number of amino acid differences per site. Partial deletion parameters were used, all positions with less than 50% site coverage were eliminated, meaning that, fewer than 50% alignment gaps, missing data, and ambiguous bases were allowed at any position. There were a total of 459 positions in the final dataset. Evolutionary analyses were conducted in MEGA6. All the branches were supported with bootstrap values ≥ 50%.

WRKY proteins are classified into 3 groups. We used phylogeny to assign a group to our WRKY sequences (Figure 2). WRKY1, 2, and 4 belong to group I. They all have 2 WRKY domains with a C-X (4,5)-C-X (22,23) H-X-H zinc-finger-like type motif on each domain. TdWRKY2 predicted protein contains two WRKY domains. The N-terminal domain has an altered WRKY motif WRKYGKK. An alignment of TdWRKY2, with its orthologous proteins from Aegilops, Brachypodium and *T.urartu* on the UniProtKB database (http://www.uniprot.org/align/), indicates that the protein is biased at its C-terminus WRKY conserved domain. 29 aa are missing from the zinc finger motif (Figure S1).

This might be the cause of alteration in Wbox recognition. The role of conserved residues in this domain has been studied by Maeo et al. (2001) using mutation experiments. Any mutation occurring either in the WRKYGQK or the zinc finger motif of WRKY domains, affecting cysteine or histidine, cancels DNA binding activity (Knoth et al., 2007). The WRKY domain from the C-terminal region is responsible for binding to DNA, whereas the role of the N-terminal is to promote protein-protein interactions (Maeo et al., 2001).

TdWRKY3 and 5 were assigned to groups IIa and IIc respectively, due to the presence of only one WRKY domain with a specific zinc finger motif, C-X (4,5)-C-X (22,25)-H-X-H.

**Figure 3:**
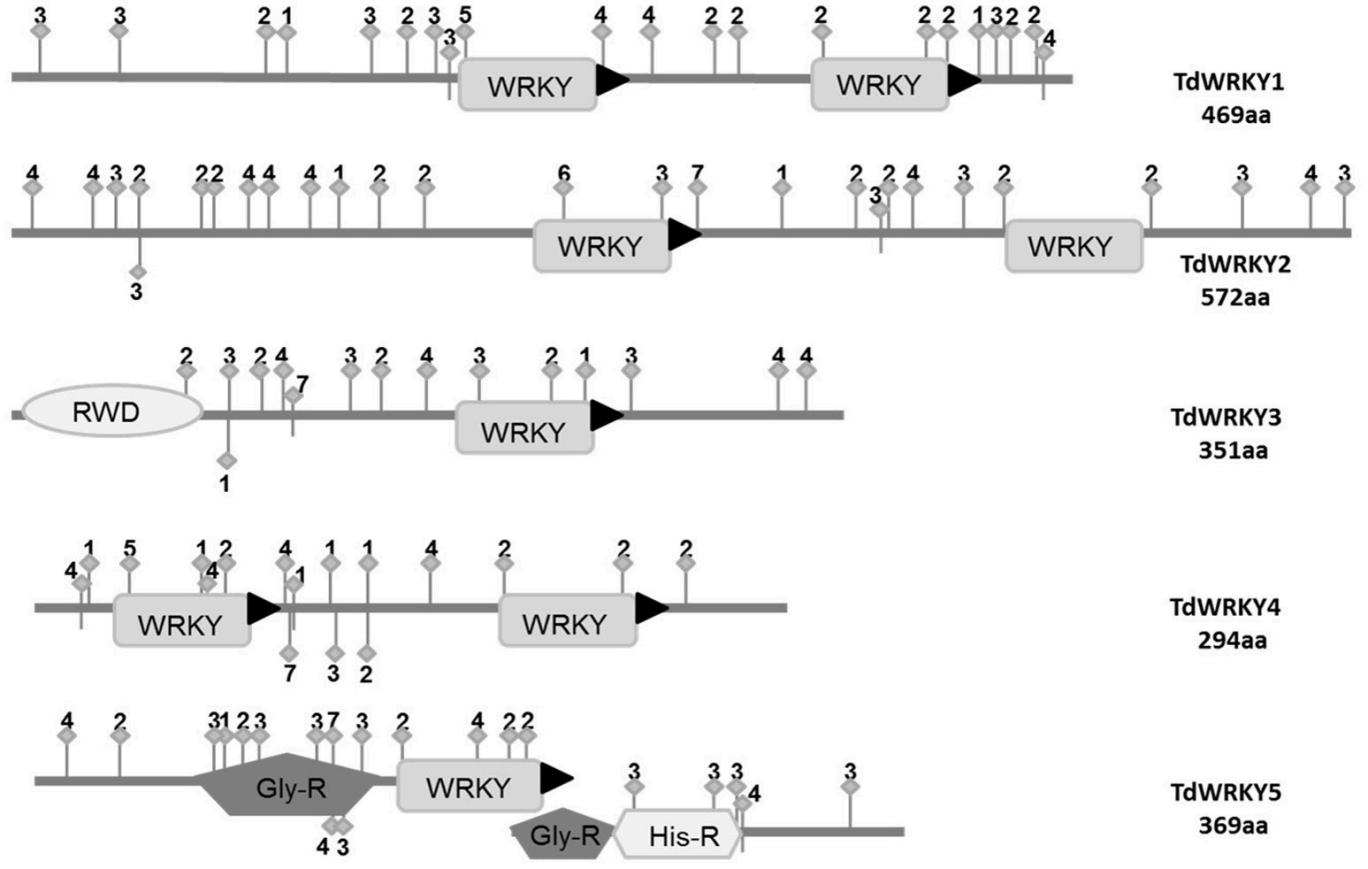
**Sequence features of the TdWRKYs protein**. Domains or motifs: WRKY (WRKY domain), RWD, Pla_Zn (Plant Zinc Clust domain), PRO_R (Pro_Rich region), MET_R (Met_Rich region), NLS (Nuclear Localization Signal), LXLXLX (repressor motif), and Fila (Filamin domain; Active sites: 1, Asn_Glycosylation; 2, CK2_Phospho_Site; 3, Microbodies_Cter site; 4, Myristyle site; 5, PKC_Phospho_Site; 6, Amidation site; 7, CAMP phosphosite).

We investigated the Prosite and Pfam databases using the TdWRKYs sequences as queries to identify conserved domains, motifs and active phosphorylation sites (Figure 3, Text S4). The analysis predicted 11 specific sequence features for all TdWRKYs identified, including the WRKY domain, a Plant Zinc Clust domain, and a Gly_Rich region and a His_Rich region (Figure 3). Seven active sites were detected, including 32 CK2_Phospho sites, 22 PKC_Phospho sites, 12 Asn_Glycosylation sites, 29 Myristyle sites, 2 TYR_pospho sites, one amidition_site and 3 CAMP_phospho_sites. These features are related to subcellular localization, signal transduction, transcriptional regulation and protein interaction, and build a basis for TdWRKY function. TdWRKY1, 4, and 5 might act as activators. TdWRKY2 and 3 contain the active repressor motif (LXLXLX) (Xie et al., 2005) (Figure 3).

For additional phylogeny support, the detection of the position and the phase of the intron in each region encoding a WRKY domain is essential. Four of the eight WRKY domains, found in *TdWRKY* genes, contain an intron in a conserved position (Figure 1). This phase 2 intron is localized at the 11th codon downstream of the WRKY motif, interrupting the codon encoding Arg (on 100% of genes). In Group Ia (*TdWRKY1, 2*, and *4*) (the C-terminal WRKY domain), Group IIc (*TdWRKY5*), IId, IIe, and III genes, this intron comes after the codons for the invariant amino acid sequence PR and separates the WRKY sequence from the zinc finger motif. In Group IIa (*TdWRKY3*) and IIb genes the intron occurs at a nucleotide position that corresponds to five amino acids after the C-X5-C and separates this from the rest of the zinc finger structure (Tripathi et al., 2012). On *TdWRKY3*, the WRKY domain was separated from the zinc finger motif by the C-X5-C core but there was not an intron at this position (Figure 1).

### Hypothetical function deduced by protein domain and orthologous gene function sequence

The phylogenetic analyses revealed distinct clusters of TdWRKYs. Within the WRKY clade, distinct clusters corresponding to WRKY groups and subgroups comprising of further sub-clusters emerged. TdWRKYs with WRKYs from wheat, barley, *T. urartu* and Aegilops formed one sub-cluster whereas rice, maize and sorghum were grouped as a separate sub-cluster, and Arabidopsis were clustered together. Within sub-clusters, durum wheat, and *T. aestivum*, WRKYs showed less divergence (as indicated by a shorter branch length) than Brachypodium and barley WRKYs. Similarly, maize and sorghum WRKYs showed less divergence compared to rice. Monocot WRKYs showed less divergence than the closest Arabidopsis WRKYs (Figure 2).

TdWRKY1 was clustered to TaWRKY19, involved in salt stress response. TdWRKY2 was clustered to BdWRKY2 and AtWRKY34, involved in cold stress response (Zou et al., 2010). TdWRKY4 belongs to the TaWRKY53, TaWRKY2 and AtWRKY33 cluster, involved in salt stress (Jiang and Deyholos, 2009). TdWRKY3 is clustered to TaWRKY80 and HvWRKY38, involved in dehydration response and to AtWRKY18, 60, and 40 (Liu et al., 2012). They were described as regulating defense response. TdWRKY5 and its orthologous TaWRKY71, BdWRKY71, and AtWRKY8, involved in salt stress response, were grouped together (Figure 2).

**Figure 4:**
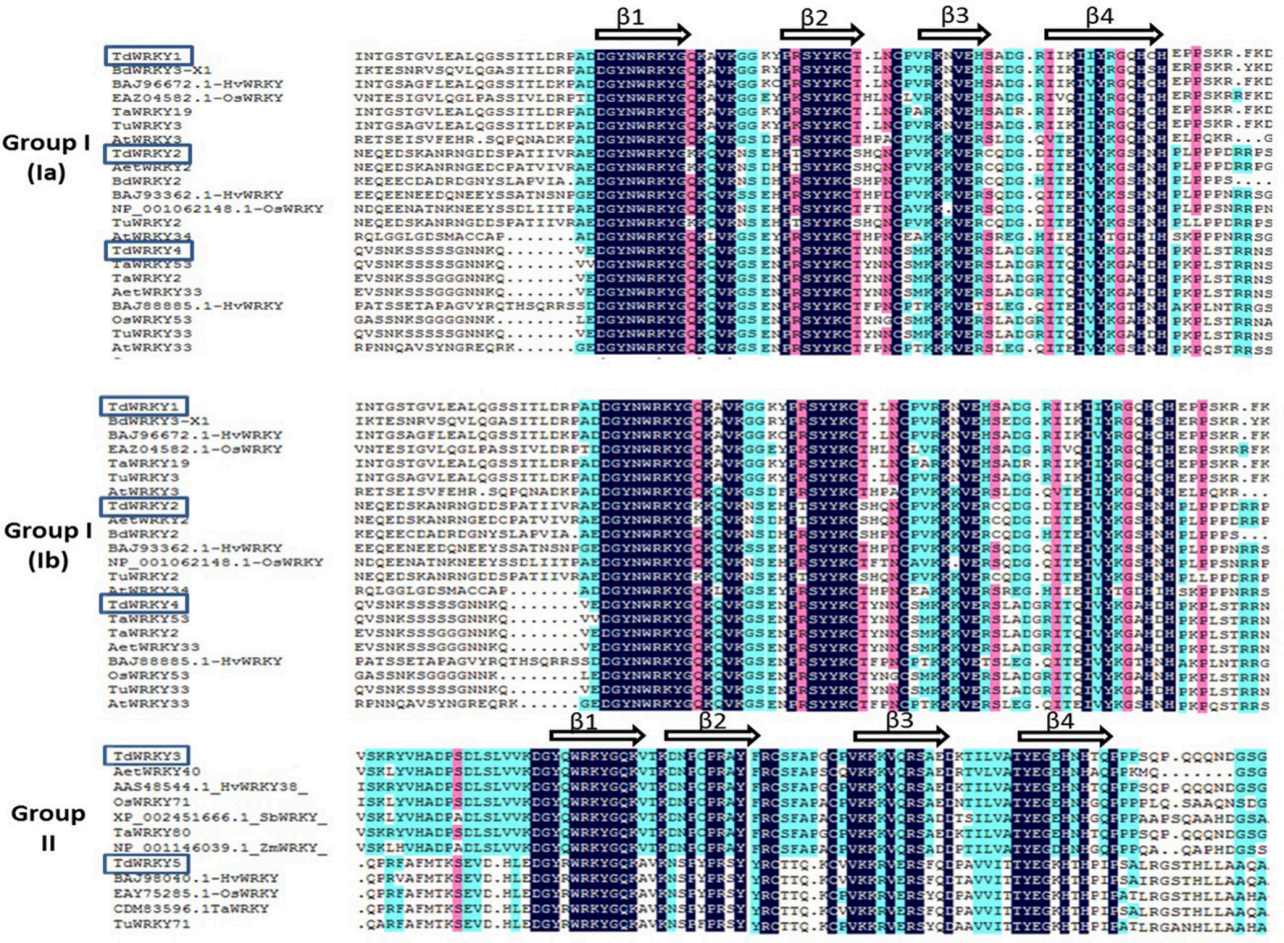
**Multiple alignment of the WRKY conserved domain of TdWRKYs with closely related WRKY proteins using DNAMAN software (http://en.bio-soft.net/format/DNAMAN.html), Blue, clear blue and pink represent 100, 75, and 50% similarity, respectively**. Four β-sheets indicated over the corresponding sequences.

Multiple sequence alignment of the conserved sequence from group Ia TdWRKYs (the two WRKY domains were aligned separately) and group IIa and IIc TdWRKY members with their closest Arabidopsis and monocot WRKY proteins (Figure 4) revealed the very conserved structure of the WRKY motifs and the amino acid residues potentially interacting with zinc ligands. TdWRKYs consist of a four-stranded ß-sheet (1, 2, 3, and 4), with a zinc binding pocket formed by the conserved Cys/His residues located at one end of the ß-sheet, and the WRKYGQK cores, corresponding to the most N-terminal ß-strand (strand ß-1), kinked in the middle of the sequence by the Glyresidue (Yamasaki et al., 2005).

### Putative cis-acting elements identified in the *TdWRKY* promoter region

BAC sequences generated more data about gene environment. It affords greater reliable and precise information for further functional studies. A 1.5 kb 5′ regulatory region upstream of the transcription start of the *TdWRKY* genes was subjected to *in silico* analysis using a plant cis-acting regulatory DNA elements (PLACE) signal scan to search for putative *cis*-regulatory elements potentially involved in the control of *TdWRKY* gene expression. The data indicated the presence of a large number of conserved *cis*-regulatory elements that are putative targets for TFs reported to mediate responses to environmental stresses or to stress-related hormones (Table 2). To regulate gene expression, WRKY factors show a binding preference to a DNA sequence called W box: (C/T) TGAC (C/T) (Ciolkowski et al., 2008). This DNA core was over-represented on *TdWRKY3, 4* and *5*. 6 and 5 boxes were found on *TdWRKY1* and *2*, respectively.

**Table 2:** **Putative cis-regulatory elements in the TdWRKY promoter regions**.

***Cis-elements***	**Sequences**	**Conditions**	**References**	**Cis-regulatory elements numbers**					**Average frequency of cis-elements in WRKY genes promoters**		
				***TdWRKY1***	***TdWRKY2***	***TdWRKY3***	***TdWRKY4***	***TdWRKY5***	***AtWRKY***	***TaWRKY***	***HvWRKY***
ABRE	YACGTGKC	ABA, dehydration, salt	Yamaguchi-Shinozaki and Shinozaki, 2005	5	0	6	6	5	0	2.2	3.4
ARF	TGTCTC	Auxin	Hagen and Guilfoyle, 2002	0	2	0	0	2	0	0	0
CBH-HV	RYCGAC	Dehydration, Cold	Svensson et al., 2006	4	0	4	0	0	0	0.6	1.4
GCC-box	GCCGCC	Ethylene, dehydration	Wu et al., 2007	5	0	0	6	0	0.73	3.8	3
LTRE	CCGAC	Cold	Baker et al., 1994	6	1	5	2	3	0	1.8	1.4
MYB	YAACKG	ABA, dehydration salt	Abe et al., 1997	12	12	11	12	12	1.47	5	6.2
MYC	CANNTG	ABA, dehydrationsalt	Abe et al., 1997	11	13	5	5	7	0	3.6	0
RAV	CAACA	Ethylene	Kagaya et al., 1999	8	9	2	4	3	0	2.8	0.4
Wbox	TGAC	Biotic and abiotic stress	Eulgem et al., 1999, 2000	6	5	14	12	15	4.4	4.2	4.6
DRE/CRT	R(A/G)CCGAC	Dehydration, Cold	Dubouzet et al., 2003; Skinner et al., 2005	4	0	3	0	1	0	1.2	2.6

*The 1.5 Kb DNA fragments corresponding to the 5′ -regulatory regions upstream of the ORF (Open Reading Frame) transcription start sites of TdWRKY genes were subjected to an in silico analysis using a PLACE signal scan and NSITE-PL to search for putative cis-regulatory motifs potentially involved in the control of TdWRKYgenes expression. R, A/G; Y, C/T; K, G/T; N, any nucleotide. Frequencies of potential binding sites in AtWRKY promoters were derived from the study of Dong et al., 2003*.

It has also been shown that some WRKY factor types can bind to other types of *cis*-elements. ABRE-like motifs (ACGTG) and ABRE-related motifs (ACGTGKC and TACGTGTC) were also found in the promoter region of *TdWRKY1 3, 4*, and *5*. The MYB-core element (TAACTG) and a number of MYB-related motifs (YAACKG, CNGTTR, and GGATG), as well as a MYC (CANNTG) motif and the MYC-related motifs (CATGTG and CACATG), were present on the entire promoters. MYC and MYB had the biggest number among others. DRE (TACCGACAT), CRT (RCCGAC), and low-temperature responsive elements (LTREs) (CCGAC), all containing the CCGAC motif that forms the core of the DRE sequence, were well represented in the promoter of *TdWRKY1 and TdWRKY3*. DRE-like elements such as CBFHv (RYCGAC) were also identified mostly on *TdWRKY1* and *TdWRKY3*. Two GCC-box motifs (AGCCGCC), target sequences for ERF proteins, were found in the *TdWRKY1 and TdWRKY4* promoters. RAV1-A motifs (CAACA), to which RAV1 proteins can bind through their AP2 and B3-like domains, were also present in a large number on *TdWRKY* promoters (Table 2). The average cis-elements frequencies on the WRKY promoter gene were deduced from *TdWRKY*s and from orthologous *TaWRKY*s and *HvWRKY*s scanned also for regulatory elements. The results showed that the promoters of *TdWRKY* genes are the richest in terms of putative regulatory elements compared to *HvWRKY*s and *TaWRKY*s. Moreover, the frequencies of GCC boxes, MYB and Wboxes in *A. thaliana* promoter regions are significantly lower than in hexaploid wheat, durum wheat and barley, which might be related to the fact that the average frequency was calculated within the 74 *WRKY* members (Dong et al., 2003). Promoter analysis in *Populus* revealed that various *cis*-acting elements (LTRE, ABRE, ABA, and Wboxes) involved in abiotic stress and phytohormone responses were highly present in the promoter region of *PtrWRKY* genes (Jiang et al., 2014). The data presented by Makhloufi et al. (2014) on durum wheat *TdERF* indicated the presence of a large number of conserved cis-regulatory elements that are putative targets for TFs reported to mediate responses to environmental stresses or to stress-related hormones.

### The expression patterns of *TdWRKY*S under salt-stress conditions in two contrasting genotypes

**Figure 5:**
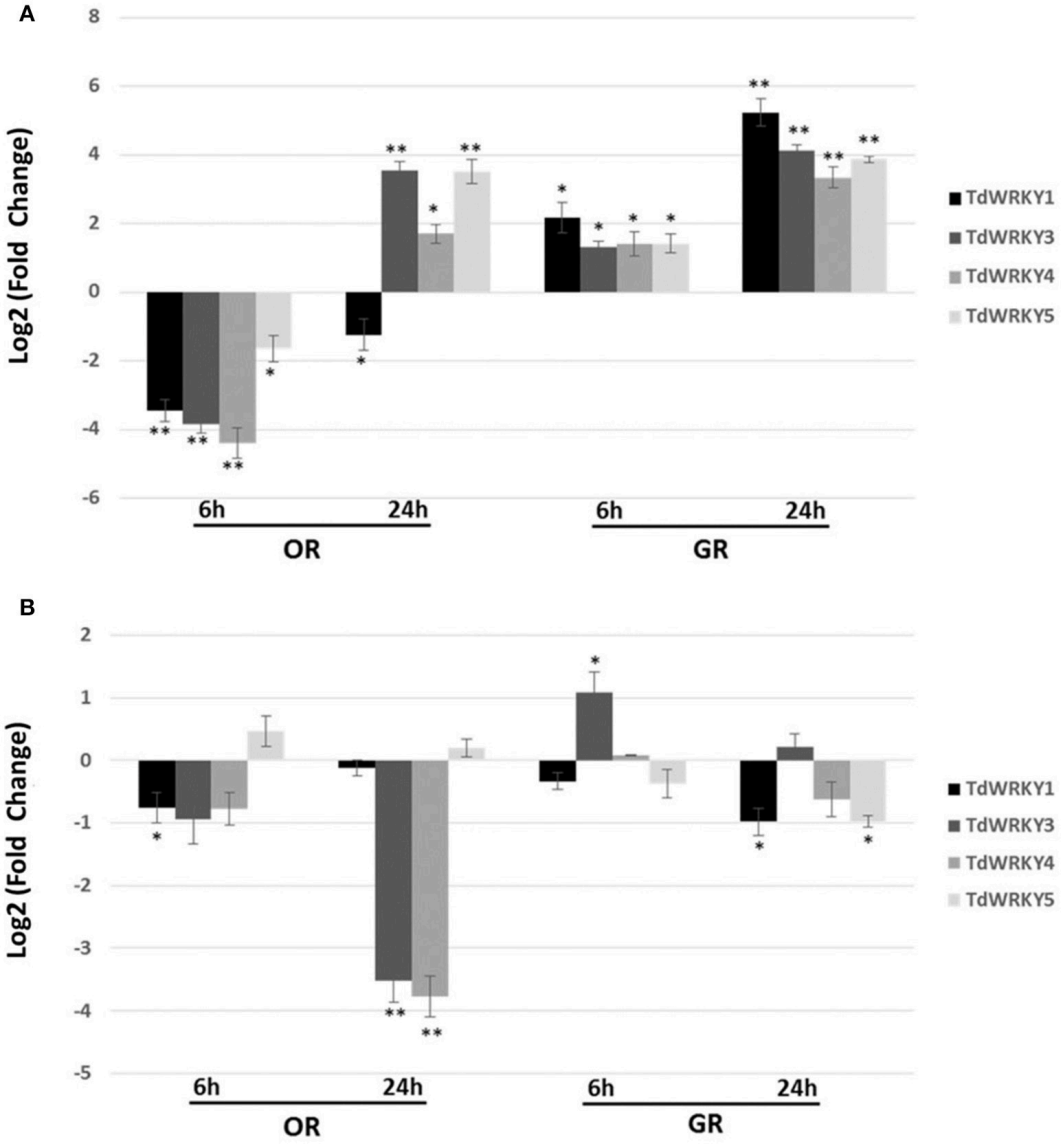
**Expression pattern of *TdWRKY1*, *TdWRKY3*, *TdWRKY4*, and *TdWRKY5* in response to salt stress**. Expression profile of *TdWRKY1, 3, 4*, and *5* in leaves **(A)** and roots **(B)** from GR-salt tolerant and OR-salt sensitive durum wheat genotypes following 200 mM NaCl treatment. The levels of *TdWRKY1, 3, 4*, and *5* transcripts were assessed by real-time quantitative PCR. *mRNA* accumulation was monitored in 10-d-old roots and leaves, after 6 and 24 h of NaCl treatment (200 mM NaCl). For each sample, relative fold changes were determined by normalizing the CT value of the *TdWRKY1, 3, 4*, and *5* genes in different tissues in both sensitive and tolerant varieties to the CT value of Td26S (internal control) and calculating the ratios of transcripts abundance compared to samples from untreated tissues, using the formula 2^−ΔΔCT^. ΔΔCT refers to fold differences in *TdWRKY1, 3, 4*, and *5* expression relative to untreated tissues. Log2-transformed signal ratio was carried out. Values are means ±SD (*n* ≥ 30 plants were pooled for RNA extraction) of three replicates. Asterisks ^*^ are used to represent *P* values: ^*^0.01 < *P* < 0.05 (Statistically significant); ^*^0.01 < *P* < 0.05; ^**^P < 0.01 (Statistically highly significant) (Student's *t*-test).

The expression pattern of *TdWRKYs* in response to short term salt stress, in both leaves and roots, was analyzed in the OR and GR genotypes of durum wheat shown to be sensitive and tolerant to high salt, respectively (Makhloufi et al., 2014). Specific primers (Table S2) were designed and used in a quantitative real-time PCR. Upon high-salt treatment (200 mM NaCl), the expression levels of *TdWRKY1, 3, 4*, and *5* were altered (up to 19-fold in *TdWRKY4*) in the leaves of sensitive genotype after 6 h under salt stress (Figure 5). Meanwhile, the transcripts level at the same time in tolerant genotype remain almost unchanged for *TdWRKY3, 4*, and 5 and increased almost 5 times than control (Figure 5A). Thereafter, the expression of the TdWRKY genes in leaf tissues displayed a dramatic increase at 24 h in both genotypes, even though the upregulation was substantially higher in GR (27-fold, 17-fold, 11-fold, and 15-fold for *TdWRKY1, 3, 4*, and *5*, respectively) than in OR (1.5-fold altered, 13-fold, 3-fold, and 12-fold for *TdWRKY1, 3, 4*, and *5*, respectively) (Figure 5A). In treated OR roots, transcripts levels of the four TdWRKYs remain unchanged at 6h in both sensitive and tolerant genotypes. Application of salt induced a decrease in transcript levels of *TdWRKY3* (12-fold) and *TdWRKY4* (14-fold) after 24 h in sensitive genotype. The expression levels of all *TdWRKY*s after 24 h of stress treatment, in tolerant genotype, remain constitutive (Figure 5B).

*TdWRKY2* carries a deletion within the fourth and last exon, just after the position encoding the second Cys of the zinc finger motif (Figure S1). By qRT-PCR analysis, we verified disruption of the gene. 2 primer pairs positioned 3′ around the deletion site did not amplify any product (Table S2). As a disruption of the zinc finger motif has been shown to completely abolish the W-box–specific DNA binding activity of WRKY transcription factors (Maeo et al., 2001), it is very likely that *TdWRKY2* is a true loss-of-function gene, as was suggested for TdWRKY2 protein.

## Discussion

### TE expansion responsible for durum wheat genome organization

Despite the accumulation of complete plant genome sequences, the most comprehensive studies on the organization of gene space throughout the sequence were carried from individual BAC clones or broader regions composed of BAC straddling, also called contigs (Brenner et al., 1993; Vitte and Bennetzen, 2006; Liu et al., 2007). We obtained, from the representative 6 BACs, a cumulative sequence length of almost 1 Mb. The TE proliferation was pronounced (representing 74.6%). LTR (Long Terminal Repeats, class I) appears to be the most represented in durum wheat sequences (63.6%) and among all grasses (Devos, 2010). Class II DNA transposons are generally less invasive than the retrotransposons in plant genomes. Durum wheat class II representation is slightly lower than bread wheat (16%) with 7.9%. Within class II members, MITE elements represent only 0.5% on durum wheat sequences; the same percentage is observed in bread wheat (Choulet et al., 2010). The composition of durum wheat is closely comparable to bread wheat composition and consequently to maize genome one (Schnable et al., 2009).

Although they are representative of the abundant wheat TEs available in the TREP database (Wicker et al., 2002, 2007; http://botserv2.uzh.ch/kelldata/trep-db/TEClassification.html), the class I and class II TEs observed in the genomic sequences of the wheat genomes may not cover all wheat TEs. It is expected that more wheat TEs will be identified, as more wheat genomic sequences become available and as more *de novo* TE annotation tools are developed (Choulet et al., 2010; Flutre et al., 2011). This is particularly supported by the identification in this study of different novel TE families, most of which are retrotransposons (17.9% of cumulative sequence).

### Durum wheat genes clustered into small islands

Our data show that durum wheat genes are clustered mainly into several very small islands (from one to four genes) per BAC, separated by large blocks of repetitive elements. Overall durum wheat gene density was estimated at one gene every 80 Kb.

Gene islands reflect a proliferation of the genome. They are common features of large and repetitive genomes, such as wheat and maize genomes and are not found in small genomes such as rice, *Arabidopsis thaliana* (The Arabidopsis Genome Initiative, 2000), and Brachypodium (Huo et al., 2009). In fact, two BAC contigs (961 and 594 kb), in maize, were analyzed and gene blocks varied between one and four genes per block (Kronmiller and Wise, 2008). This suggests that within large plant genomes, gene islands may originate from a specific selection against the separation of genes by TE insertions that would be deleterious for gene expression or regulation and second a homogeneous expansion combined with preferential deletions in gene-rich regions (Choulet et al., 2010).

Genes from islands would share common functional characteristics (Hurst et al., 2004). Genes are maintained close to one another because this configuration would provide a selection advantage and functional significance (Batada et al., 2007; Janga et al., 2008). This is confirmed by the identification of co-expressed genes or relatives sharing the same functions that are conserved between the Arabidopsis, rice and poplar genomes (Liu and Han, 2009). *TdWRKY2* and *gene2* coding for a chloride channel protein (CLC) (Table 1) (from TD16L16), involved in vacuolar compartmentation during salt stress (Hechenberger et al., 1996), might share the same function. This assumption is more defensible, because of their very close genomic distance; they are separated only by 500 bp. The same gene structure is conserved on *B. distachyon* orthologous region. Gene ontology and expression profiles along the chromosome in *T. aestivum* revealed that these islands are enriched in genes sharing the same function or expression profiles suggesting the existence of long-distance regulation mechanisms in wheat (Choulet et al., 2010).

### Correlation between sequence similarity and functional similarity

Protein sequences contain important information for protein function. We found that TdWRKYs have sequence features including domains/motifs: WRKY domains, which bind WRKY proteins to the W-box motif in the promoter of target genes for transcription regulation; Plant Zinc finger motifs which function in association with WRKY domains; NLS (nuclear Localization Signal) peptides, responsible for leading proteins to the cell nucleus. And active sites such as protein kinases, which play important roles in signal transduction pathways. Special features such as Myristyl sites could function during TdWRKY5-mediated gene responses to stresses, as myristoylation sites play a vital role in membrane targeting and signal transduction in plant responses to environmental stresses (Podell and Gribskov, 2004).

Homologous genes with similar sequences are likely to have equivalent functions and to play the same functional role in equivalent biological processes. It is thus very important to identify homologous genes, especially those which are supported by experimental data. TdWRKYs from different groups (Ia, IIa, and IIc) have a close relationship with orthologous genes from barley, Brachypodium, bread wheat, maize, sorghum, aegilops, *T. urartu*, rice and Arabidopsis. Genes from *T. aestivum, Ae. tauschii, T. urartu*, and *H. vulgare* shared the closest similarity, while rice, sorghum and maize were out-grouped from other monocot plants.

As summarized in Table 2, the promoter regions of TdWRKYs were highly rich in cis-acting elements, and most of them were related to stress-induced gene expression, suggesting the putative role of essentially TdWRKY1, 3, 4, and 5 genes in wheat responses to a variety of environmental stresses. A TBLASTN on NCBI nucleotide collection database of the *TdWRKY*s 1.5 Kb cis-elements showed that *TdWRKY1*-UTR region shares 92% similarity from base 1 to 419 bp with *T. aestivum* 3B genome scaffold HG670306.1 from 28534545576 to 285345992. This 419 bp contains 4 W Box, 4 MYB, 5MYC, 4RAV, and 1 ABRE elements. 1.5 Kb *TdWRKY2-*UTR (632-949) region also shares 84% similarity with *HG670306.1* (74734822-74734532). Elements which might be common to the orthologous regions are 3 Wbox, 4MYB, and 4MYC. 100% of homology was found between the 1.5 Kb from *TdWRKY5* putative *cis-*elements and its homolog *HG670306* from coordinate 436276014 to coordinate 436275955. They share all the cis-elements on Table 2. Finally, 99% was shared between all UTR regions from *KC174859.1* (*TaWRKY53*) and 1.5 Kb from 736 to 1501. This region contains 4 Wbox, 6 MYB, 1 MYC, 4 GCC cores, 2 RAV, 2 LTREs, and 3 ABREs. Two hypotheses might be formulated. Firstly, genes that share the same acting elements on their promoters may function the same way and, secondly, homologous *cis*-acting elements of *TdWRKY1, TdWRKY2*, and *TdWRKY4* are assigned to the 3B chromosome in bread wheat, suggesting that *TdWRKY1, 2*, and *4* might be also accommodated within chromosome 3B or 3A from *T. turgidum*.

Promoter region analyses, gene structure within orthologous members, and phylogeny studies all indicated that *TdWRKY*s are novel members of the WRKY family in durum wheat and, given their high sequence homology with orthologous monocots and Arabidopsis' known function, it can be postulated that they play similar roles in mediating responses to biotic and abiotic stresses.

### Differential expression of *TdWRKY*S

In a context where no functional characterization has yet been carried out for WRKYs in durum wheat, our data showed that the *TdWRKY* genes were inducible by high-salt treatment. Moreover, *TdWRKY* expression in response to salt stress displayed distinctive patterns in two durum wheat genotypes with contrasting behavior regarding tolerance to abiotic stresses. In the tolerant GR variety, *TdWRKY*s were strongly induced by salt stress within a few hours (6 h), while it was downregulated in the sensitive OR variety. Notably, differences between tolerant and sensitive genotypes were detected, mainly in the expression levels in tolerant genotype leaves at 24 h stress treatment. Peng et al. (2014) showed that *WRKY* members' unigenes were mostly up-regulated under salt stress in cotton (*Gossypium hirsutum* L.). They noted that some *WRKY* genes were expressed in the salt-tolerant genotype Earlistaple 7, but were repressed, weakly induced, or not induced at all in salt-sensitive Nan Dan Ba Di Da Hua, within 24 h. *HvvWRKY2* was induced by salt stress in TR1 (Tolerant variety) but not in TS1 (Sensitive variety) (Li et al., 2014). Similarly, expression of wheat *TaWRKY2* and *TaWRKY19* was induced by salt, and both *TaWRKY2* and *TaWRKY19* enhanced salt tolerance in transgenic Arabidopsis plants compared with wild type (Niu et al., 2012). The induction pattern showed that the highest gene expression occurs at 3–6 h after salt stress initiation, for *TaWRKY2* and *TaWRKY19* and at 6 and 24 h for *HvvWRKY2*, which is likely to be the same for *TdWRKY* members.

## Concluding remarks

The durum wheat complexity and large genome size (13,000 Mb) have largely prevented the development of genomic resources. Meanwhile, efforts have focused on sequencing of target regions selected as covering one or more genes of interest, called locus. Several BAC clones covering the corresponding region in one or more wheat genomes were sequenced as well.

In this study, we targeted durum wheat BAC clones harboring *TdWRKY* genes potentially involved in response to salt stress. We validated six BAC clones relative to the genomic library TtuLDN65. The size of these clone inserts after sequencing varies between 120 and 190 Kb. The added value of such an approach is that we obtain the coding sequence with introns and promoter regions that are essential for expression or functional study. Furthermore, it enabled us to access the entire genomic environment of the coding sequence that might provide new information about the structure, conservation, position, order and genomic dynamics. A structural study of the environment of a gene encoding a resistance or tolerance protein can bring a multitude of information that can enrich functional study.

In this study, we identified 5 *WRKY* genes potentially involved in the salt stress tolerance. This article reports the identification of 5 novel *TdWRKY* genes. Sequence comparison with orthologs from barley, wheat and Brachypodium provide valuable information for determining gene structure. *TdWRKY1, TdWRKY3, TdWRKY4*, and *TdWRKY5* gene sequences were highly conserved as well as exon-intron boundaries, even with Arabidopsis. These important structural similarities, between orthologous *WRKY* genes, are indicative of a potential functional conservation.

## Author contributions

HB, MB, AG initiated the project. FY, EM, WM performed experiments. FY performed analysis and interpretation of data for the work. FY wrote the paper. HB, WM revised the paper critically for important intellectual content.

### Conflict of interest statement

The authors declare that the research was conducted in the absence of any commercial or financial relationships that could be construed as a potential conflict of interest.

## Abbreviations

ABRE: ABA-responsive element
BAC: bacterial artificial chromosome
CRT: C-repeat element
DRE: dehydration-responsive element
ERF: ethylene response factor
EST: expressed sequence tag
LTRE: low-temperature-responsive element
NLS: nuclear localization signal
ORF: open reading frame
TE: transposable element
TF: transcription factor
UTR: untranslated region.
