# Supplementary material for: Comparative Analysis of WRKY Genes Potentially Involved in Salt Stress Responses in Triticum turgidum L. ssp. durum
Source: Front Plant Sci. 2017 Jan 31;7:2034. doi: 10.3389/fpls.2016.02034 (PMC5281569; doi:10.3389/fpls.2016.02034)
Supplement: Supplementary file 1 [file DataSheet1.DOCX]

***Supplementary Material***

**Characterization of *WRKY* genes potentially involved in salt stress responses in *Triticum turgidum* spp *durum***

**Fatma-EzzahraYousfi^1,2,3^, Emna Makhloufi^1,2,3^, William Marande^4^, AbdelWahed Ghorbel^3^, Mondher Bouzayen^1,2^ and Hélène Bergès^4*^**

1 University of Toulouse, INPT, Laboratoire de Genomique et Biotechnologie des Fruits, Avenue de l’Agrobiopole BP 32607, Castanet-Tolosan F-31326, France

2 INRA, UMR990 Genomique et Biotechnologie des Fruits, Castanet-Tolosan, F-31326, France

3 Center of Biotechnology of BorjCedria (CBBC).Lab. Plant Molecular Physiology.BorjCedria Science and Technology Park - B.P.901,Hammam-lif 2050, Tunisia

4 Centre National de Ressources GenomiquesVegetales, INRA–CNRGV, 24 Chemin de Borde Rouge, CS 52627, 31326 Castanet-Tolosan, France

* To whom correspondence should be addressed. E-mail: Helene.Berges@toulouse.inra.fr

**Table S1.** Primers design for durum wheat BAC screening and isolation of *TdWRKY*s sequence

| **Putatif TdWRKY** | **Primers name** | **Sequence (5’->3’)** | **Source** | **(bp)** |
| --- | --- | --- | --- | --- |
| ***TdWRKY2*** | EST2-2 F | AAGCACGTCGAGAGGGCGTC | *AJ717156*  *Genebank* | 275 |
|  | EST2-2 R | CGCGGGGCAGCATTCCAAAG |  | 275 |
|  | EST2-5 F | CCACCGCCATCGGTACTAT |  | 152 |
|  | EST2-5 R | AAGTTGTCAAAGGCAACCCA |  | 152 |
| ***TdWRKY5*** | EST3-2 F | CGCAGAAGTGTGTGGTGAA | *AJ613825*  *Genebank* | 164 |
|  | EST3-2 R | GTGGTGCTGGTGATGCAG |  | 164 |
| ***TdWRKY1*** | TaWRKY19-1F | CAGGACAACTTTGATGTGTTCCA | *EU665430*  *Genebank* | 288 |
|  | TaWRKY19-1R | TCCAGTGTTGACACTACCACT |  | 288 |
|  | TaWRKY19-2F | ATGCCAAGGAGCCTTCAACT |  | 200 |
|  | TaWRKY19-2R | TGCATCGGCATCACCATCAT |  | 200 |
| ***TdWRKY3*** | HvWRKY38-1F | GTTGCGGTTCTTGAGTCGGA | *AY541586*  *Genebank* | 396 |
|  | HvWRKY38-1R | GCTGAGGTCGGAGGGGT |  | 396 |
| ***TdWRKY4*** | TaWRKY2-1F | TCCACGGGGAGCTTGGA | *EU665425*  *Genebank* | 325 |
|  | TaWRKY2-1R | AGGAGAACGGGGGAGTCG |  | 325 |
|  | TaWRKY2-3F | AGGAGCTACTACAAGTGCACAA |  | 170 |
|  | TaWRKY2-3R | GAGGAAGATGACGTCGGCAG |  | 170 |

**Table.S2**. Primers design for quantitative Real Time-PCR

| **Noms des amorces** | **Séquences (5’- 3’)** | **Sources** | **Longueur des fragments amplifiés (pb)** |
| --- | --- | --- | --- |
| ***TdWRKY1-F*** | GGTCAACACCACTGCAAAGAG | TD14H23 | 105 |
| ***TdWRKY1-R*** | GAGATCGTTCAGAATGGCTGT |  |  |
| ***TdWRKY2-1F*** | ATCCATCGGCTATGCAGG | TD16L16 | 100 |
| ***TdWRKY2-1R*** | ACCAGTTCCACTCTGGTCT |  |  |
| ***TdWRKY2-2F*** | GATTATGTCACTCCAGCCACC | TD16L16 | 100 |
| ***TdWRKY2-2R*** | GCTTGTGGTCTGCACGAT |  |  |
| ***TdWRKY3-F*** | TGATGTCCCTGGTCGGC | TD473J1 | 101 |
| ***TdWRKY3-R*** | GCAGATGGCCATGACGC |  |  |
| ***TdWRKY4-F*** | AGGATCATACCAACGGCTCG | TD493B21 | 105 |
| ***TdWRKY4-R*** | CGGCGCTTGGTCTCGG |  |  |
| ***TdWRKY5-F*** | CGCAGAAGTGTGTGGTGAA | BAC315C7 | 101 |
| ***TdWRKY5-R*** | GTGGTGCTGGTGATGCAG |  |  |
| ***26S-F*** | GAGTCGTTTGGCACCTCGAT | [AP008982\|](http://www.ncbi.nlm.nih.gov/nucleotide/78675232?report=genbank&log$=nuclalign&blast_rank=4&RID=EMRUGDRG014) | 101 |
| ***26S-R*** | AACCCAACTCACGTACCACTTTC |  |  |

**Text.S1.** Annotation details

TriAnnot allows: first, TEs annotation through the REPET package developed by Quesneville et al. (2005), a classical similarity search performed by RepeatMasker against the TREP databank (Wicker et al., 2002) and “in-house” annotated TEs (Choulet et al., 2010). Second, through masking TEs by using the k-mer composition to mask repeated regions (Leroy et al., 2012). The next panel of BAC sequences annotation is dedicated to structural and functional annotation of protein-coding genes; the Exon–intron junctions and the protein coding sequences (CDS) are predicted *ab initio*, by sequence similarity, or through the two approaches combined. TriAnnot uses four programs for *ab initio* gene prediction: FGeneSH17, GeneID, GeneMark.HMM and Augustus (Leroy et al., 2012). A similarity search (BLASTP) against several protein databanks and against the Pfam protein domain collection with HMMER 3.018, assign putative function to Gene models. Comparative sequence analysis of genomic regions from related species can greatly support gene identification in the annotation process. Triannot allows also the identification of non-coding *RNA* genes through tRNAscan and conserved non-coding sequences using BLASTN/BLASTX search similarities against major plant genomes (*Arabidopsis*, Oryza, Zea, Sorghum, Brachypodium). Finally, because of their high density within wheat sequences (one SSR every 13.1 kb) (Choulet et al., 2010), TriAnnot uses the TRF program (Tandem Repeats Finder; Benson, 1999) for marker design (Leroy et al., 2012).

**Text.S2.** Composition of the durum wheat Intergenic space

TEs prediction, annotation, classification were performed according to the unified classification system for eukaryotic TEs (Leroy et al., 2012).The 74.6% of TEs space were shown to be composed of a wide variety of TEs, distributed as follows: 63.6% for class I, 7.9% for class II and 3.1% for unclassified TEs (Supplementary table S4).While class I retrotransposons constitute the highest TEs proportion of the 6 sequenced regions, BAC clone TD473J1 shows the highest proportion of *CACTA* class II (17.0 %).

The 63.6% class I TEs covered 612,343Kb. Two main retrotransposon superfamilies constitute the majority of class I TE DNA sequences, as follows: 27,2% *Gypsy*- (150 TEs) and 15.9% *Copia-* (71 TE) like ‘long terminal repeats (LTR)’-retrotransposons (Table S4). Class I TEs composition, range from 36.2% to 71.5%, depending on the sequenced region BAC. New class I transposable element were identified for the first time in this study. They account for 17.9% by length and were identified as *de nono* LTR-retrotransposon and for16.1%by length also, as *de novo* long interspersed nuclear elements (LINEs). The novel LTR retrotransposons show some similarities with *Copia*-like and others with known *Gypsy* retrotransposon families. They were designated as *de novo*, based on the TE classification guidelines (Wicker *et al.* 2007), and considered as belonging to the same super-families of the referenced TEs with which they show the highest similarity.

Class II TEs DNA transposons represent 7.9 % of the cumulative sequence length (141 TEs) and are composed of 47*CACTA*, 25 MITEs and LITEs. In term of sequence representation, the *CACTA* TEs represent the majority (32.4%) of class II DNA sequences. As in previous studies with *Triticeae* (Wicker *et al.*, 2005) the *CACTA* transposons were often found clustered in the genome. This is particularly the case for BAC clone TD473J1 where 26 *CACTA* TEs (complete and truncated) were found representing 17 % of the 147, 9 kb (Table S4). The other five BAC clones are relatively *CACTA*-poor regions containing 0 to 9 *CACTA* TEs representing only 1.0% of the whole sequence of BAC TD906L16.52 novel class II TE families were identified, sharing weak homologies with known *CACTA, Mutator* and *Mariner*. The 3.1% novel unclassified element, share a stretch of weak homology with other *Triticeae* unclassified transposable elements

|  |  | TD14H23 | | | TD16L16 | | | TD789O23 | | | TD493B21 | | | TD473J1 | | | TD315C07 | | | Total | |
| --- | --- | --- | --- | --- | --- | --- | --- | --- | --- | --- | --- | --- | --- | --- | --- | --- | --- | --- | --- | --- | --- |
| **Superfamily** | | **Size (pb)** | **Nb** | **(%)** | **Size (pb)** | **Nb** | **(%)** | **Size (pb)** | **Nb** | **(%)** | **Size (pb)** | **Nb** | **(%)** | **Size (pb)** | **Nb** | **(%)** | **Size (pb)** | **Nb** | **(%)** | **Size(pb)** | **(%)** |
| ***Classe I*** |  |  |  |  |  |  |  |  |  |  |  |  |  |  |  |  |  |  |  |  |  |
| ***LTR*** | ***Gypsy*** | 54215 | 17 | 31,6 | 39334 | 22 | 32,4 | 39884 | 25 | 23,8 | 71090 | 55 | 37,2 | 19962 | 14 | 13,5 | 36419 | 27 | 22,6 | 260904 | 27,2 |
|  | ***Copia*** | 27185 | 8 | 16 | 33445 | 16 | 27,5 | 33473 | 18 | 20,0 | 24443 | 13 | 12,8 | 13245 | 7 | 9,0 | 20840 | 23 | 12,9 | 152631 | 15,9 |
|  | ***Unknown*** | 27123 | 17 | 15,8 | 5868 | 7 | 4,8 | 36425 | 16 | 21,8 | 29006 | 18 | 15,1 | 19501 | 10 | 13,2 | 54090 | 15 | 33,5 | 172013 | 17,9 |
| ***LINE*** | ***Unknown*** | 9898 | 9 | 6 | 852 | 6 | 0,7 | 9110 | 6 | 5,4 | 1377 | 8 | 0,7 | 1223 | 3 | 0,8 | 4334 | 7 | 2,7 | 26794 | 16,1 |
| ***Classe II*** |  |  |  |  |  |  |  |  |  |  |  |  |  |  |  |  |  |  |  |  |  |
| ***TIR*** | ***Mariner*** | 115 | 1 | 0,07 | 406 | 4 | 0,3 | 286 | 3 | 0,2 | 0 | 0 | 0,0 | 247 | 2 | 0,2 | 0 | 0 | 0,0 | 1054 | 0,1 |
|  | ***Mutator*** | 1287 | 5 | 0,8 | 0 | 0 | 0,0 | 976 | 1 | 0,6 | 49 | 1 | 0,0 | 462 | 2 | 0,3 | 781 | 2 | 0,5 | 3555 | 0,4 |
|  | ***CACTA*** | 265 | 4 | 0,2 | 1268 | 9 | 1,0 | 1735 | 3 | 1,0 | 0 | 0 | 0,0 | 25174 | 26 | 17,0 | 873 | 5 | 0,5 | 29315 | 3,1 |
|  | ***Unknown*** | 6461 | 9 | 3,8 | 208 | 2 | 0,2 | 1595 | 8 | 1,0 | 9223 | 6 | 4,8 | 16750 | 19 | 11,3 | 2962 | 8 | 1,8 | 37199 | 3,9 |
| ***MITE*** |  | 1400 | 7 | 0,8 | 1361 | 8 | 1,1 | 256 | 1 | 0,2 | 0 | 0 | 0,0 | 0 | 0 | 0,0 | 1495 | 5 | 0,9 | 4512 | 0,5 |
| **Unclassidfied** | | 28716 | 11 | 16,7 | 178 | 1 | 0,0 | 0 | 0 | 0,0 | 0 | 0 | 0,0 | 290 | 1 | 0,2 | 1077 | 1 | 0,7 | 30261 | 3,1 |
| **Total ETs size (pb)** | | 156665 |  | 91% | 82920 |  | 68,3 | 123740 |  | 73,9 | 133811 |  | 70,0 | 95631 |  | 64,7 | 118537 |  | 73,4 | 711304 | 74,0 |
| **Total BAC insert size (pb)** | | 171472 |  |  | 121491 |  |  | 167361 |  |  | 191051 |  |  | 147902 |  |  | 161492 |  |  | 960769 |  |

**Table.S3**. Classification and distribution of known and unknown durum wheat TEs from BAC sequences

**Text.S3.**Gene prediction and density of the durum wheat genomic region and gene structure

Analysis of the 12 genes and fragment models revealed a gene size ranging from 683bp to 6.843 kb with an average size of 3094.4bp, an average number of 3.5 exons per gene and 25% of genes without introns.  41% of the genes contained two or three exons, whereas, 16 % had 9 exons comparable to what has been described for maize (Haberer et al., 2005). The average coding sequence (CDS), from cumulative six BACs sequence 960.769Kb, is 1135 bp, which is close to the average value (1143 bp) obtained from 6137 full-length cDNAs from wheat (Mochida et al., 2009), indicating that our sample provides a good representation for the durum wheat genes. Intron sizes were highly variable ranging from 80bp to 2.514 kb with an average size of 1971 bp clearly higher than 130 pb median size observed in bread wheat (Choulet et al., 2010), also higher than that observed in rice (median 138 bp; Yu et al., 2002), and in maize introns (median 166 bp; Haberer et al., 2005).

Unknown repeated DNA sequences represent 3.1 % among the 74.6 % of TEs and unassigned DNA sequences account for 22.2 % of the cumulative sequence length. The remaining 3.2 % are related to gene islands.

A gene prediction and analysis of the 3.2 % non-TEs and non-repeated DNA was conducted using different search programs. Exon–intron structures and the protein coding sequences (CDS) were predicted and putative function for the gene models are assigned via a combination of similarity search (BLASTP) against several protein databanks and against the Pfam. TriAnnot follows a nomenclature based on the guideline established in 2006 by the IWGSC annotation.

Genes of known or putative function were defined on the basis of 80% and over 45%, respectively, with known proteins in UniProtKB/Swiss-Prot.Hypothetical genes were identified on the basis of prediction programs only; when no similarity is found, either in UniProtKB/Swiss-Prot or UniProtKB/TrEMBL, or Pfam domain or ESTs. Conserved-unknown function genes are defined when no expressed sequence is found, and when *>*45% similarity over *>*50% of the protein length is found only with an unknown function in UniProtKB/Swiss-Prot and UniProtKB/TrEMBL. Domain-containing-protein hasn’t significant BLASTP hit with a known or putative function but one or more Pfam domains (Leroy et al., 2012). The expressed sequence protein are defined based on TBLASTN against plant EST databanks with *>*45% identity and *>*50% coverage. Pseudogenes are not predicted because TriAnnot does not annotate pseudogenes automatically. Combined together all these types of gene sequence information (GSI) accounting for only 3.2 % of the sequence are present in six BAC clones (one gene per clone for TD14H23, TD473J1 and TD493B21)while the remaining two BAC clones (TD16L16 and TD789O23),contain four genes (Table S6).Two genes (of known or unknown function), three genes with putative function, four genes with domain containing protein, one hypothetical gene and one truncated (fragmented gene), were identified (Table S6).

The genomic sequence of the first *TdWRK*Y1 gene (from TD14H23) length was 3534 bp with a total size for the predicted *cDNA* of 1762 bp and a complete open reading frame of 1407 bp. The length of the predicted 5’-untranslated region (UTR), exon 1, intron 1, exon 2, intron 2, exon 3, intron 3, exon 4 and 3’UTR were 68, 189, 927, 842, 107, 117, 738, 259 and 287 bp, respectively (Figure 1). The predicted protein TdWRKY1 has a high level of similarity with the known function involved in salt stress *TaWRKY19* protein (98% similarity, Table1) and with the closest Arabidopsis homologue (NP_178433, 45%similarity). *TaWRKY19* and *TdWRKY1* genes are divided in four exons with identical size (Figure 1). Exon 3 and 4 has identical size in all four *Poaceae*species and exon 1has identical length in Brachypodium, durum wheat and *T.aestivum.* However exon 1 from *HvWRKY* (AK365469.1) has 20 amino acids more than the predicted durum wheat protein at the start codon. Analysis of the second exon of durum wheat predicted protein, showed that it has the same length as the *TaWRKY19* (EU665430). Although, the overall similarity in length between the four second exons, exon 2 is divided into two more exons in *BdiWRKY3.1* (XM_003562717.3) and in three more exons in *HvWRKY* (AK365469.1), which possess a fifth exon more than all others (Figure 1).

The genomic sequence of the *TdWRKY3* gene (from TD473J1) was 4295 bp with a total size for the predicted *cDNA* of 4186 bp and a complete open reading frame of 1053 bp. The length of the predicted 5’-untranslated region (UTR), exon 1, intron 1, exon 2, and 3’UTR were 1907, 156, 109, 897 and 1226 bp (Figure 1). The putative protein from durum wheat TdWRKY3 (from TD473J1) also has a high level of similarity with the putative *T.aestivum* protein (100% similarity, Table 1) and less similarity with the closest Arabidopsis homologue (NP_178199.1, 36%similarity, Table 1).*TdWRKY3* shares 92% and 80% similarity in *cDNA* sequence with *HvWRKY* (AK360269.1)and *BdiWRKY40* (XM_003570693.3), respectively. *TdWRKY* is divided in 2 exons: the first exon is identical between durum wheat and *B.distachyon*. This same exon1 has similar length as exon 3 in *H.vulgarec* DNAwhich has two more exons The second exon of the durum wheat gene showed similarity to the last two exons from *H.vulgare* and its length (210 bp) was identical to the sum of the lengths of the last three exons in *B.distachyon* (57 and 153 bp) (Figure 1). The predicted gene structure in durum wheat has been confirmed by the alignment of the predicted *cDNA* based on the genomic sequence with *Hvwrky38* (AY541586.1, 94%identical) (Table 1). The presence of this sequence in *cDNA* library related to salt stress may provide an indication of its function.

*TdWRKY4* (from TD493B21) is the smallest *WRKY* gene compared with others *TdWRKY*s, with 294 aa amino. The full length genomic sequence size is 1227 bp. The full length 980 bp predicted *TdWRKY4 cDNA* is composed of 5’UTR, exon 1, intron 1, exon 2, intron 2, exon 3 and 3’UTR with 0, 419, 135, 171, 112, 292 and 98 bp, respectively*. TdWRKY4*’s CDS size is 882bp.The putative function translated protein shows 98% similarity with TaWRKY53 (AGF90798.1); already involved in salt stress (Niu et al., 2012) (Table 1) and are both divided in 3 exons, one of them (exon 2) of identical length (Fig. 1B). The closest Arabidopsis homologue is AtWRKY33 (AAM34736.1, 54% similarity with durum wheat predicted protein, Table 1). Short stretches of non-homology between *T.aestivum* and durum wheat cDNAs exon 1 were found 77bp upstream of the start codon of *TaWRKY53*. *T.aestivum* exon 3 stretches 98bp downstream of durum wheat exon 3 stop codon.Predicted BdiWRKY ( from PACid: 21813358)  exons 5 and 6 are identical in length compared to durum wheat exon 1 and 2, annotation shows 123bp stretch on durum wheat exon 3 compared to its exon 7 homolog from *B.distachyon*. ButPACid: 21813358 has four additional predicted exons upstream to durum wheat start codon (Figure 1).

Full length 2157bp novel *TdWRKY5* (from TD315C07) genomic sequence has neither 5’ nor 3’ untranslated regions. Predicted introns sizes are 915 and 135 bp for intron 1 and intron 2. Predicted full length 1107 bp*cDNA* exon 1, 2 and 3 sized: 398, 162 and 547 bp, respectively (Figure 1).An analysis of the colinear durum wheat, *Triticumaestivum*, barley and Brachypodium, shows that the predicted *cDNA*s from durum wheat has 99% similarity (Table 1) with predicted *T. aestivum* “HG670306.1” from (*Triticumaestivum* chromosome 3B, genomic scaffold, cultivar Chinese Spring). It shares 92% identity with *HvWRKY* -NIASHv1067K13 (AK358052.1). *TdWRKY* and its homologs in *T.aestivum* and *B.distachyon* are divided in 3 exons. The 3 exons are identical in length between *B.distachyon* and *T.durum* homologous genes (Figure 1). The exon1 in *T.aestivum* is slightly shorter than exon 1 from *TdWRKY* and bread wheat exon 3 is slightly longer than *TdWRKY* exon3. The four homologuous genes share the same structure for exon 2. Exon 3 is divided in 2 more exons, which make *HvWRKY*-NIASHv1067K13 have a more additional exon than others collinear *cDNA*s, which is 97bp downstream longer. Standard GT/AG, exon-intron boundaries found were highly conserved between the four *Poaceae* species, suggesting conservation in their biological roles.

|  |  | **BLASTP** | | | | | **BLASTn** | | | | |
| --- | --- | --- | --- | --- | --- | --- | --- | --- | --- | --- | --- |
| **Clone BAC** | ***T.turgidum* predicted gene** | **Protein Acc.No** | | **Identity** | **Score** | **Expect** | **cDNA Acc.No** | | **Identity** | **Score** | **Expect** |
| **TD14H23** | **Known_function, WRKY DNA binding domain**  **3534bp**  **1407bp**  **469aa**  **CAT01** | *T.aestivum* | ACD80362.1 | 98% | 953 | 0.0 | *T.aestivum* | EU665430.1 | 99% | 2521 | 0.0 |
|  |  | *T.urartu* | EMS57536.1 | 94% | 811 | 0.0 | *-* | - | - | - | - |
|  |  | *H.vulgare* | BAJ96672.1 | 78% | 755 | 0.0 | *H.vulgare* | AK365469.1 | 93% | 1625 | 0.0 |
|  |  | *B.distachyon* | XP_003562765.1 | 70% | 635 | 0.0 | *B.distachyon* | XM_003562717.3 | 86% | 1137 | 0.0 |
|  |  | *A.thaiana* | NP_178433.1 | 45% | 228 | 1e-67 | *-* | - | - | - | - |
| **TD16L16** | **Putative_function, Putative WRKY transcription WRKY**  **3052bp**  **1716bp**  **572aa**  **CAT04** | *T.urartu* | EMS51071.1 | 93% | 1124 | 0.0 | *B.distachyon* | XM_010236958.1 | 86% | 522 | 5e-69 |
|  |  | *A taushii* | EMT16491.1 | 88% | 1010 | 0.0 | *-* | - | - | - | - |
|  |  | *B.distachyon* | XP_010235260.1 | 53% | 536 | 2e-180 | *-* | - | - | - | - |
|  |  | *A.thaliana* | NP_200438.1 | 30% | 199 | 9e-55 | *-* | - | - | - | - |
|  | **Domain_containing_protein, Voltage gated chloride channel**  **6134bp**  **2232bp**  **744aa**  **CAT01** | *B.distachyon* | XP_010235261.2 | 94% | 1368 | 0.0 | *B.distachyon* | XM_010236959.2 | 99% | 2663 | 0.0 |
|  |  | *S.italica* | XP_004973814.1 | 89% | 1285 | 0.0 | *O.sativa* | NM_001068682.1 | 99% | 2303 | 0.0 |
|  |  | *S.bicolor* | XP_002445942.1 | 88% | 1273 | 0.0 | *O.sativa* | AK120828.1 | 99% | 2287 | 0.0 |
|  |  | *O.brachyantha* | XP_006659542.1 | 89% | 1267 | 0.0 | *S.italica* | XM_004973757.1 | 99% | 2217 | 0.0 |
|  |  | *O.sativa* | NP_001062147.1 | 90% | 1259 | 0.0 | *O.brachyantha* | XM_006659479.1 | 98% | 2148 | 0.0 |
|  |  | *A.thaliana* | NP_564698.1 | 67% | 938 | 0.0 |  |  |  |  |  |
|  | **Domain_containing_protein, Pentatricopeptide, PPR**  **1347bp**  **449aa**  **CAT02** | *B.distachyon* | XP_003574702.1 | 84% | 778 | 0.0 | *B.distachyon* | XM_014900254.1 | 86% | 1402 | 0.0 |
|  |  | *O.sativa* | NP_001062150.1 | 81% | 744 | 0.0 | *S.italica* | XM_004973758.2 | 83% | 1227 | 0.0 |
|  |  | *O.brachyantha* | XP_006659545.1 | 80% | 731 | 0.0 | *O.sativa* | AP014964.1 | 83% | 1117 | 0.0 |
|  |  | *T.urartu* | EMS53378.1 | 99% | 729 | 0.0 | *Z.mays* | NM_001175976.1 | 81% | 1066 | 0.0 |
|  |  | *S.italica* | XP_004973815.1 | 78% | 712 | 0.0 | *S.bicolor* | XM_002445894.1 | 83% | 1031 | 0.0 |
|  |  | *A.thaliana* | NP_178132.1 | 53% | 416 | 6e-142 |  |  |  |  |  |
|  | **Domain_containing_protein, Acetyltransferase (GNAT) family**  **4806 bp**  **1476 bp**  **492aa**  **CAT02** | *O.sativa* | EAZ27421.1 | 92% | 894 | 0.0 | *T.turgidum* | FR820620.1 | 98% | 1786 | 0.0 |
|  |  | *Z.mays* | XP_008658770.1 | 89% | 887 | 0.0 | *O.sativa* | NM_001056961.1 | 88% | 1727 | 0.0 |
| **TD493B21** |  | *S.bicolor* | XP_002467646.1 | 89% | 872 | 0.0 | *O.brachyantha* | XM_006651459.1 | 87% | 1687 | 0.0 |
|  |  | *A taushii* | EMT10107.1 | 97% | 774 | 0.0 | *S.bicolor* | XM_002467601.1 | 86% | 1605 | 0.0 |
|  |  | *H.vulgare* | BAJ92090.1 | 81% | 764 | 0.0 | *S.italica* | XM_004983861.2 | 86% | 1592 | 0.0 |
|  |  | *T.urartu* | EMS53376.1 | 98% | 733 | 0.0 | *Z.mays* | XM_008660548.1 | 86% | 1555 | 0.0 |
|  |  | *A.thaliana* | AAC32438.1 | 65% | 651 | 0.0 |  |  |  |  |  |
|  | **Putative function, WRKY DNA binding domain**  **1227bp**  **882bp**  **294aa**  **CAT01** | *T.aestivum* | ABN43185.1 | 99% | 605 | 0.0 | *T.aestivum* | EF368364.1 | 99% | 1591 | 0.0 |
|  |  | *T.aestivum* | AGF90798.1 | 98% | 602 | 0.0 | *S.italica* | XM_004962359.3 | 79% | 503 | 3e-138 |
|  |  | *A.tauschii* | EMT33135.1 | 87% | 508 | 2e-176 | *T.aestivum* | EF368357.1 | 92% | 1151 | 0.0 |
|  |  | *A.thaliana* | AAM34736.1 | 54% | 278 | 5e-89 | *T.aestivum* | EU665443.1 | 91% | 1138 | 0.0 |

**Table.S4.** Triticum durum BAC clones Triannot gene models and orthologous comparative sequences from Blastp and Blastn

**Table.S4.** Triticum durum BAC clones Triannot gene models and orthologous comparative sequences from Blastp and Blastn

|  |  | **BLASTP** | | | | | **BLASTn** | | | | |
| --- | --- | --- | --- | --- | --- | --- | --- | --- | --- | --- | --- |
| **BAC clone** | ***T.turgidum* predicted gene** | **Protein Acc.No** | | **Identity** | **Score** | **Expect** | **cDNAc Acc.No** | | **Identity** | **Score** | **Expect** |
| **TD473J1** | **Putative_function, WRKY transcription factor**  **4295bp**  **1053bp**  **351aa**  **CAT04** |  |  |  |  |  |  |  |  |  |  |
|  |  | *T.aestivum* | AFW98256.1 | 100% | 724 | 0.0 | *T.aestivum* | JX679079.1 | 99% | 1940 | 0.0 |
|  |  | *H.vulgare* | CAD60651.1 | 91% | 626 | 0.0 | *H.vulgare* | AK360269.1 | 92% | 1487 | 0.0 |
|  |  | *S.purpurea* | AJF34891.1 | 81% | 554 | 0.0 | *H.vulgare* | AY541586.1 | 94% | 1367 | 0.0 |
|  |  | *B.distachyon* | XP_003570741.1 | 70% | 437 | 3e-149 | *B.distachyon* | XM_003570693.3 | 80% | 734 | 0.0 |
|  |  | *A,thaliana* | NP_178199.1 | 36% | 198 | 9e-60 |  |  |  |  |  |
| **TD315C07** | **Domain_containing_protein, WRKY DNA binding domain** | *T.aestivum* | CDM83596.1 | 100% | 751 | 0.0 | *H.vulgare* | AK358052.1 | 92% | 1507 | 0.0 |
|  | **2157bp** | *H.vulgare* | BAJ98040.1 | 89% | 511 | 1e-177 | *T.aestivum* | HG670306.1 | 99% | 1011 | 0.0 |
|  | **1107bp** | *B.distachyon* | XP_003569516.1 | 70% | 356 | 1e-116 | *B.distachyon* | XM_003569468.3 | 82% | 861 | 0.0 |
|  | **369aa** | *A. thaliana* | NP_174279.1 | 46% | 173 | 3e-50 |  |  |  |  |  |
|  | CAT02 |  |  |  |  |  |  |  |  |  |  |
| **TD789O23** | **Troncated gene , WRKY DNA binding domain (gene1)** |  |  |  |  |  |  |  |  |  |  |
|  | **683bp** | *T.aestivum* | CDM83596.1 | 98% | 285 | 3e-85 | *T.aestivum* | HG670306.1 | 93% | 990 | 0.0 |
|  | **402bp** | *H.vulgare* | BAJ98040.1 | 91% | 177 | 4e-51 | *H.vulgare* | AK358052.1 | 92% | 785 | 9e-162 |
|  | **134aa** | *B.distachyon* | XP_003569516.1 | 74% | 100 | 4e-22 | *B.distachyon* | XM_003569468.3 | 78% | 246 | 4e-61 |
|  | CAT04 |  |  |  |  |  |  |  |  |  |  |
|  | **Troncated gene , WRKY DNA binding domain (gene4)** | *T.aestivum* | CDM83596.1 | 98% | 375 | 1e-125 | *H.vulgare* | AK358052.1 | 93% | 1016 | 0.0 |
|  | **1567bp** | *H.vulgare* | BAJ98040.1 | 91% | 352 | 8e-117 | *T.aestivum* | HG670306.1 | 94% | 1689 | 0.0 |
|  | **861bp** | *B.distachyon* | XP_003569516.1 | 74% | 273 | 2e-85 | *B.distachyon* | XM_003569468.3 | 84% | 584 | 9e-163 |
|  | **287aa** | *A.thaliana* | NP_193551.1 | 75% | 174 | 5e-51 |  |  |  |  |  |
|  | CAT03 |  |  |  |  |  |  |  |  |  |  |
|  | **Conserved unknown function, Ribokinase** | *H.vulgare* | BAJ94855.1 | 97% | 391 | 5e-133 | *T.aestivum* | HG670306.1 | 98% | 1123 | 0.0 |
|  | **1488bp** | *B.distachyon* | XP_003569514.1 | 93% | 365 | 6e-123 | *H.vulgare* | AK361328.1 | 97% | 1000 | 0.0 |
|  | **660bp** | *S.italica* | XP_004969456.1 | 82% | 329 | 1e-108 | *B.distachyon* | XM_003569466.3 | 92% | 845 | 0.0 |
|  | **220aa** | *Z.mays* | AFW83487.1 | 80% | 307 | 2e-102 |  |  |  |  |  |
|  | **CAT04** | *A.thaliana* | NP_001077551.1 | 54% | 202 | 2e-62 |  |  |  |  |  |
|  | **Hypothetical gene** | No similarity | | | | | No similarity | | | | |
|  | **CAT04** |  | |  |  |  |  | |  |  |  |

**Text.S4.** Protein special features

Biological functions of genes are closely dependent from sequence features. Therefore, we investigated the Prosite and Pfam databases using the TdWRKYs sequences as queries to identify conserved domains, motifs and active sites. The analysis predicted 11 sequence features for all TdWRKYs. Two WRKY conserved domains were predicted for group I members: TdWRKY1, TdWRKY2 and TdWRKY4. We identified only one WRKY domain for group II members TdWRKY3 and TdWRKY5, which contains also a Plant Zinc domain at its N-terminal moiety. A Gly_Rich and His_Rich regions were shown on TdWRKY5. Glycine-rich RNA-binding proteins (GRPs) were found to be involved in post-transcriptional regulation of salt stress response (Wang et al., 2011) (Figure3).

Signals from the outside of a cell are mediated to the inside of that cell by protein–protein interactions of the signaling molecules. The activity of DNA binding of some WRKY proteins may be dependent on their phosphorylation status (Yang et al., 1999; Yamamoto et al., 2004).

Seven active sites were detected, including: 2, 2, 2, 5 and 1 Asn_Glycosylation sites (1) (Asn-X(2)-Ser/Thr) (Gavel et Von Heijne, 1990) in TdWRKY1, 2, 3, 4 and 5, respectively.  9, 9, 4, 5 and 5 CK2_Phospho_Sites (Casein kinase II) were associated to predicted TdWRKY1, 2, 3, 4 and 5, too. Casein kinase II is involved in the regulation of gene transcription through its ability to phosphorylate transcription factors. Nieva et al. 2005 concluded that CK2 protein in maize plays an important role in response to ABA produced in response to water stress through the phosphorylation of two bZIP transcription factors known for stress signaling regulation. The third type of sites found is the N-Myristoylation one (G- {EDRKHPFYW} –x(2)-[STAGCN]- {P} is the consensus sequence and G is the N-myristoylation site) (Towler et al., 1988). Six sites have been deduced from TdWRKY1, 7 from TdWRKY2, 4 from TdWRKY3, 2 from TdWRKY4 and finally 9 from TdWRKY5. These active sites enhance the association of the protein to plasma membrane which makes the requirement of N-myristoylation for SOS3 function, a proof that the transporter is directly regulated at the plasma membrane. They are also required for protein/protein interaction (Figure 3).

Another post traductional TdWRKYssites are the PKC_Phospho_Sites (4) (Protein kinase C). Three and seven active PKC sites were revealed in TdWRKY2 and 1, respectively. Four PKC sites were also revealed on each protein sequence of TdWRKY3, 4 and 5. Tyrosine kinase phosphorylation sites were not abundant as others sites. We detected only one site on TdWRKY1 and on TdWRKY4 protein. No similar site consensus has been detected on the other durum wheat proteins. Tyrosine kinase was found to be involved in salt stress response in Arabidopsis (Ulm et al., 2002). It also mediates and regulates stomatal movement(Luan, 2002). Amidation site (AMIDITION) (6) was only found on TdWRKY2 protein. Finally, two CAMP_PHOSPHO_SITE (cAMPdependant protein) with [RK](2)-x-[ST] consensus have been deduced from TdWRKY2 and TdWRKY4 (Figure 3).

All the Putative phosphorylation sites, qualified the five TdWRKYs as a potential phosphorylation substrates for one or more protein kinases.

Subcellular localization experiments (transient transformation of tobacco protoplasts or onion cells) showed the forwarding of these proteins to the nucleus (Eulgem et al., 2000). Transportation in the nucleus may also depend on the interaction with other proteins.

The deduced peptide sequences were searched for the consensus coactivator motif (LXXLL) and the active repressor motif (LXLXLX) where L is Leu and X is any amino acid, to predict whether TdWRKYs proteins have putative activating activity or repressing one (Xie et al., 2005). TdWRKY1, 4 and 5contain the coactivator motif. TdWRKY2 and 3 contain the active repressor motif.

Multiple sequence alignment of the conserved sequence from group Ia TdWRKYs (the two WRKY domains were aligned separately) and group IIa and IIc TdWRKYs members with their closest Arabidopsis and monocots WRKY proteins (Fig. 4) revealed a very conserved structure of the WRKY motifs and the amino acid residues potentially interacting with zinc ligands. TdWRKYs consists of a four-stranded ß-sheet (1, 2, 3 and 4), with a zinc binding pocket formed by the conserved Cys/His residues located at one end of the ß-sheet, and the WRKYGQK cores, corresponding to the most N-terminal ß-strand (strand ß-1), kinked in the middle of the sequence by the Gly residue (Yamasaki et al., 2005).
