# Supplementary figures and images for: Comparative Analysis of WRKY Genes Potentially Involved in Salt Stress Responses in Triticum turgidum L. ssp. durum
Source: Front Plant Sci. 2017 Jan 31;7:2034. doi: 10.3389/fpls.2016.02034 (PMC5281569; doi:10.3389/fpls.2016.02034)

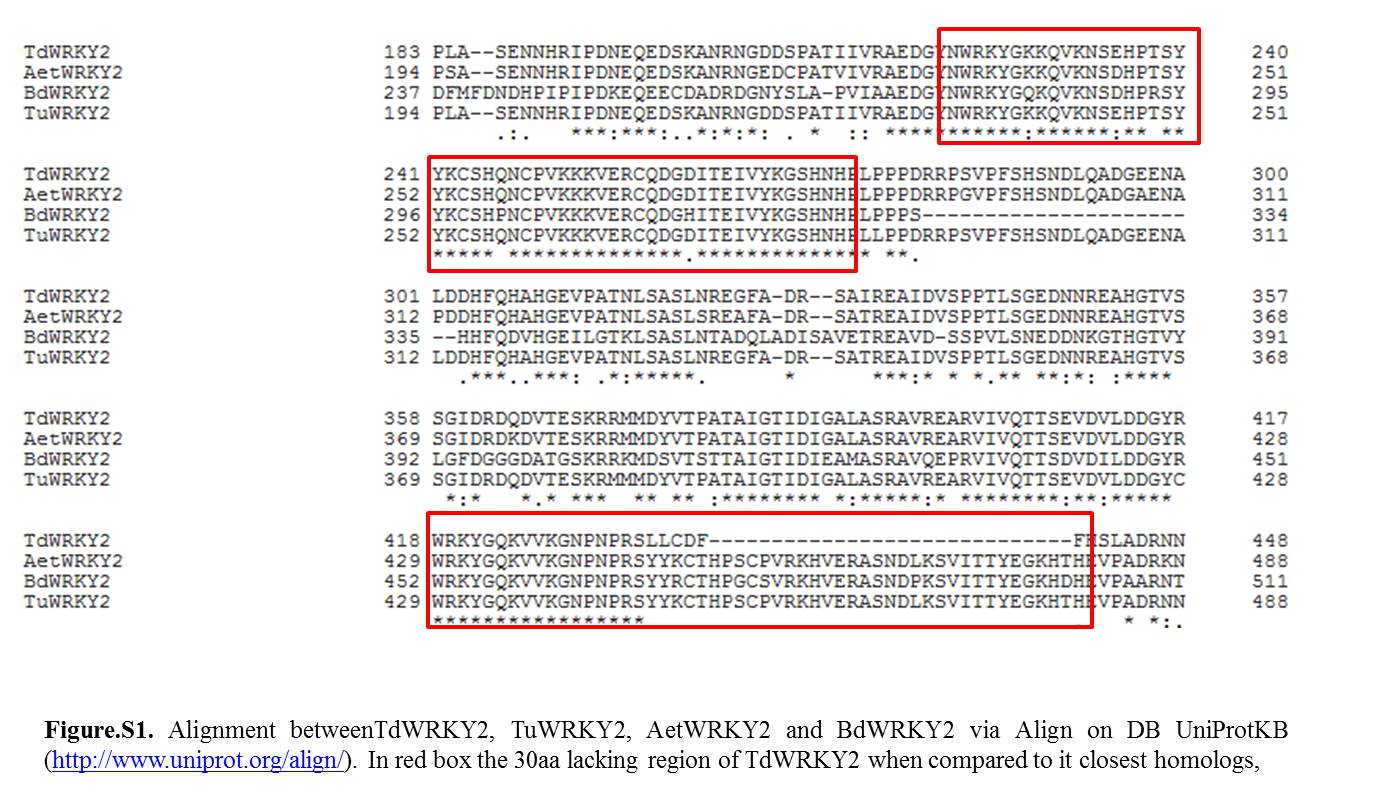

Supplement: Supplementary file 3 [file Image1.JPEG]

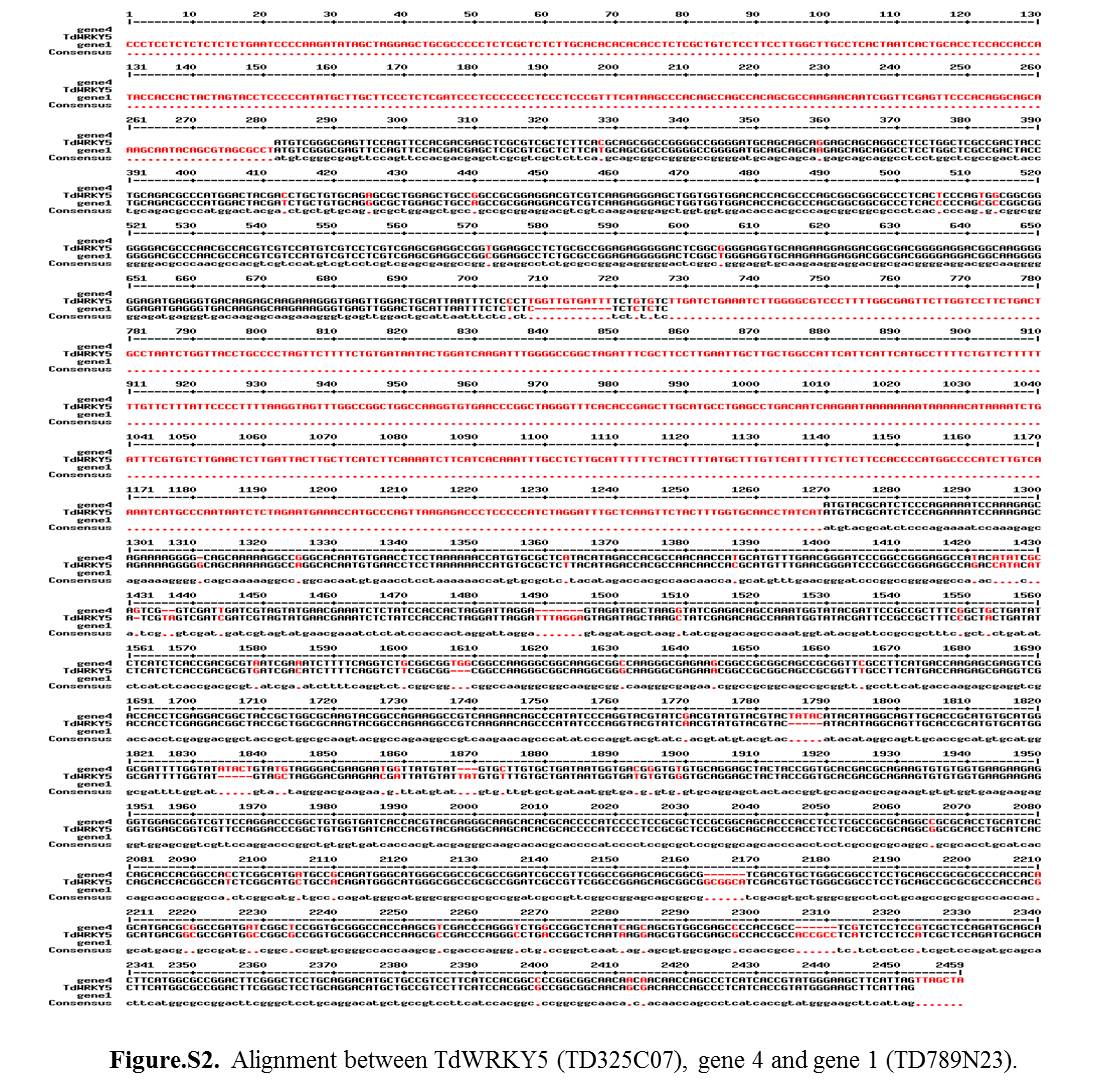

Supplement: Supplementary file 4 [file Image2.JPEG]
